# A novel missense variant in ACAA1 contributes to early-onset Alzheimer’s disease, impairs lysosomal function, and facilitates amyloid-β pathology and cognitive decline

**DOI:** 10.1038/s41392-021-00748-4

**Published:** 2021-08-31

**Authors:** Rongcan Luo, Yu Fan, Jing Yang, Maosen Ye, Deng-Feng Zhang, Kun Guo, Xiao Li, Rui Bi, Min Xu, Lu-Xiu Yang, Yu Li, Xiaoqian Ran, Hong-Yan Jiang, Chen Zhang, Liwen Tan, Nengyin Sheng, Yong-Gang Yao

**Affiliations:** 1 Key Laboratory of Animal Models and Human Disease Mechanisms of the Chinese Academy of Sciences & Yunnan Province, and KIZ-CUHK Joint Laboratory of Bioresources and Molecular Research in Common Diseases, Kunming Institute of Zoology, Chinese Academy of Sciences, Kunming, Yunnan China; 2 Kunming College of Life Science, University of Chinese Academy of Sciences, Kunming, Yunnan China; 3 State Key Laboratory of Genetic Resources and Evolution, Kunming Institute of Zoology, Chinese Academy of Sciences, Kunming, China; 4 Department of Psychiatry, The First Affiliated Hospital of Kunming Medical University, Kunming, China; 5 Division of Mood Disorders, Shanghai Mental Health Center, Shanghai Jiao Tong University School of Medicine, Shanghai, China; 6 Mental Health Institute of the Second Xiangya Hospital, Central South University, Changsha, China; 7 Center for Excellence in Animal Evolution and Genetics, Chinese Academy of Sciences, Kunming, Yunnan China; 8 CAS Center for Excellence in Brain Science and Intelligence Technology, Chinese Academy of Sciences, Shanghai, China

**Keywords:** Neurological disorders, Molecular neuroscience

## Abstract

Alzheimer’s disease (AD) is characterized by progressive synaptic dysfunction, neuronal death, and brain atrophy, with amyloid-β (Aβ) plaque deposits and hyperphosphorylated tau neurofibrillary tangle accumulation in the brain tissue, which all lead to loss of cognitive function. Pathogenic mutations in the well-known AD causal genes including *APP*, *PSEN1*, and *PSEN2* impair a variety of pathways, including protein processing, axonal transport, and metabolic homeostasis. Here we identified a missense variant rs117916664 (c.896T>C, p.Asn299Ser [p.N299S]) of the acetyl-CoA acyltransferase 1 (*ACAA1*) gene in a Han Chinese AD family by whole-genome sequencing and validated its association with early-onset familial AD in an independent cohort. Further in vitro and in vivo evidence showed that ACAA1 p.N299S contributes to AD by disturbing its enzymatic activity, impairing lysosomal function, and aggravating the Aβ pathology and neuronal loss, which finally caused cognitive impairment in a murine model. Our findings reveal a fundamental role of peroxisome-mediated lysosomal dysfunction in AD pathogenesis.

## Introduction

Alzheimer’s disease (AD, MIM: 104300) is a devastating neurodegenerative disease that afflicts a large portion of the aged population at an ever increasing rate. Synaptic dysfunction, neuronal loss, amyloid plaques (main component amyloid-β (Aβ) peptide), tau inclusions (main component hyperphosphorylated tau), brain atrophy, and cognitive impairment are pathological and clinical features of AD.^1,2^ Accumulating evidence showed that both genetic and environmental factors affect AD, and its heritability has been estimated to be very high (up to 0.79).^2–4^ The genes involved in the Aβ production, such as *APP* (Aβ precursor protein), *PSEN1* (Presenilin-1), and *PSEN2* (Presenilin-2), were identified as the causal genes for some cases with early-onset familial AD (EOFAD) more than three decades ago.^5–11^ However, most of the pathogenic mutations of these causal genes presented in an autosomal-dominant manner and only occurred in a low proportion of (<5%) of AD patients.^5,12^ It has been shown that AD is polygenic, with many causal and/or risk genes remain to be identified.^3,13–15^ Over 40 well-confirmed AD risk loci have been reported in genome-wide association analyses (GWAS) of late-onset AD, with the APOE e4 allele being the most influential factor.^13,14,16,17^ Most of these GWAS loci are common single-nucleotide polymorphisms located in non-coding genomic regions, with unknown function annotation and a small-to-moderate effect sizes (odds ratio [OR] <1.2). In fact, only 16% of the total AD phenotypic variance has been attributed to these GWAS hits,^15,18^ while other risk variants, especially these functionally causative variants^14,15^ in unknown genes and epigenetic alterations,^19,20^ still show some promise of helping our understanding of the complex genetic structure of AD. For instance, we recently found a missense variant p.K420Q in complement C7 to be associated with AD in Han Chinese.^21^

Over 50 loci/genes involved in a variety of pathways, including endocytosis, cholesterol and lipid metabolism, synaptic function, dendritic and axonal transport, Aβ and tau processing, and microglial and myeloid cell function, have been implicated in AD,^14,22,23^ suggesting that AD is a systemic disease.^24^ There are multiple reports for dysfunction of metabolism during the AD pathogenesis.^25–28^ In this study, we reported an EOFAD-associated rare loss-of-function variant, rs117916664 (p.Asn299Ser [p.N299S]), in peroxisomal *ACAA1* (acetyl-CoA acyltransferase 1). The ACAA1 p.N299S results in loss of function of the ACAA1 enzyme and impairs the lysosomal function, disturbs global gene expression pattern, affects cellular function, and controls the expression network in human AD. Overexpression of ACAA1 p.N299S in an AD mouse model facilitates Aβ pathology and exacerbates neurodegeneration. Our results demonstrate that ACAA1 p.N299S significantly aggravates Aβ pathologies and Aβ-mediated neurodegeneration, supporting a role of loss of function of ACAA1 as a risk factor for AD development.

## Results

### Association of ACAA1 p.N299S with EOFAD in Han Chinese

**Fig. 1:**
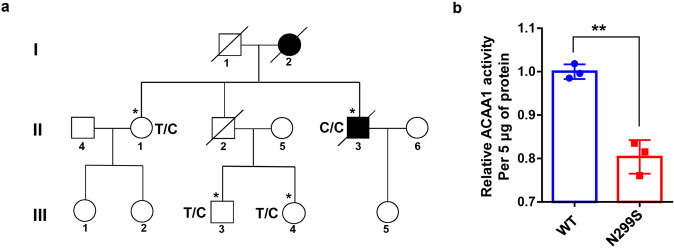
The *ACAA1* c.896T>C (p.N299S) variant identified in a Han Chinese pedigree with familiar AD disturbed acyltransferase activity. **a** Pedigree of a Han Chinese family with AD. Individuals who underwent whole-genome sequencing are indicated by asterisks (*). The subject with heterozygous or homozygous allele of rs117916664 was marked by T/C or C/C in the pedigree. **b** ACAA1 p.N299S protein (N299S) has reduced enzyme activity compared to the wild-type ACAA1 (WT). Purified ACAA1 p.N299S and ACAA1 WT were used for detection of acetyltransferase activity, with ACAA1 WT as the reference for normalization (*n* = 3 biological replicates for each group). Results are mean ± SD. **P < 0.01, Student’s *t* test

We enrolled an EOFAD pedigree in Han Chinese from Southwest China (Fig. 1a) and performed whole-genome sequencing (WGS) on samples from four individuals in this family: the proband (II:3, male, 57 years) and his mother (I:2, female) had AD, but his sister (II:1, 63 years), one nephew (III:3, 34 years), and one niece (III:4, 27 years) were non-AD. No pathogenic mutations (rare damaging variants) in the three AD-causal genes *APP*, *PSEN1*, and *PSEN2* or other neurodegenerative disorder-causal genes were observed in the AD proband or in other individuals of this family (Supplementaty Table S1). As there might be novel pathogenic mutation(s) accounting for the onset of EOFAD in this pedigree, we looked for rare (with a minor allele frequency [MAF] ≤0.01 in the dataset of the 1000 Genomes Project^29^) and potentially damaging variants (including missense, nonsense, and frameshift variants). We identified a total of 58 rare potentially damaging variants and APOE ε4 in the four individuals (Supplementaty Table S2). The three non-AD family members had the APOE ε4 allele, but the AD proband was APOE ε4 negative and had nine potentially damaging variants (ACAA1 p.N299S, TET2 p.E1151*, TBC1D3D p.K25*, PSG4 p.Y351*, OR4X2 p.Y27*, SLC6A18 p.Y319*, GEMIN8 p.E195V, DMD p.K1510R, and GPR112 p.P368H); each variant had a genotype different from that of other non-AD members (Supplementaty Table S2).

In order to investigate which variant might be associated with EOFAD, we screened for these 58 potentially damaging variants in the whole-exome sequencing data of 169 patients with EOFAD that were reported in our previous studies.^21,30^ The *ACAA1* variant (GenBank: NM_001130410.1: c.896T>C, rs117916664, p.N299S; PHRED-scaled Combined Annotation-Dependent Depletion (CADD) score^31^ 19.72), which was homozygous in the AD proband (Fig. 1a), was significantly enriched in EOFAD patients (MAF = 0.0525) compared to controls (MAF = 0.0204) (OR = 2.662, *P* value = 9.85 × 10^−3^). The other eight damaging variants were either absent in the exome data^21,30^ or showed no association with AD (Supplementaty Table S2). Note that, in European populations,^32,33^ variant ACAA1 p.N299S has been shown to be extremely rare (MAF < 0.01%), indicating a population-specific effect.

### Variant p.N299S impaired enzymatic activity of ACAA1

The *ACAA1* is named as peroxisomal 3-oxoacyl-coenzyme A thiolase or 3-ketoacyl-CoA thiolase, peroxisomal. Mutation p.N299S occurs in an evolutionarily conserved residue in ACAA1 (Supplementaty Fig. S1). As *ACAA1* is a member of the acetyl-CoA acyltransferase family and plays an important role in fatty acid β-oxidation of the very-long-chain fatty acid (VLCFA),^34^ we compared the enzymatic activities of wild-type (WT) ACAA1 and mutant p.N299S by an in vitro enzymatic activity assay.^35^ The enzymatic activity of ACAA1 p.N299S is lower than that of ACAA1 WT protein (Fig. 1b), indicating that ACAA1 p.N299S is a loss-of-function variant causing a reduction in enzymatic activity.

### ACAA1 p.N299S disturbed lysosomal and synaptic function

**Fig. 2:**
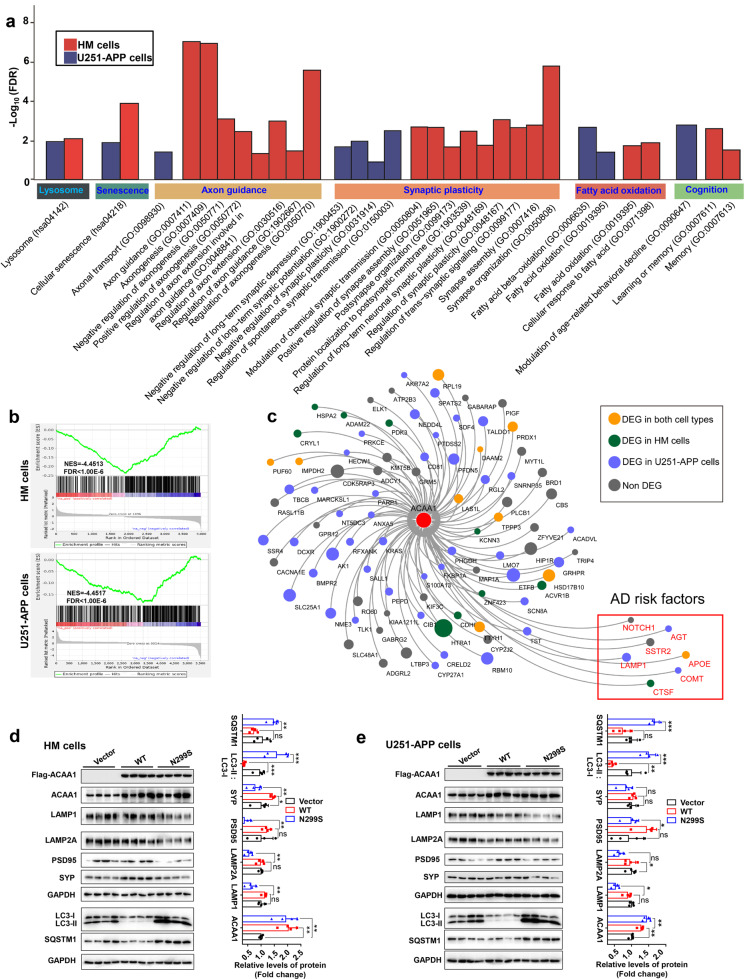
Overexpression of ACAA1 p.N299S disturbed global gene expression pattern and inhibited lysosomal and synaptic proteins in human cells. **a** KEGG pathway and GO biological processes analyses of differentially expressed genes in the HM and U251-APP cells overexpressing ACAA1 p.N299S and ACAA1 WT. **b** Enrichment of neuron development genes in cells with overexpression of ACAA1 p.N299S versus ACAA1 WT (upper, HM cells; below, U251-APP cells) based on gene set enrichment analyses (GSEA). **c** Overexpression of ACAA1 p.N299S versus ACAA1 WT affects co-expression network constructed using human AD brain tissues. **d**, **e** Overexpression of ACAA1 p.N299S in HM (**d**) and U251-APP cells (**e**) reduced the levels of lysosomal and postsynaptic proteins and increased LC3-II:LC3-I ratio and SQSTM1 protein level. The GAPDH was used as the loading control. Data are representative of three independent experiments with similar results. Bars represent mean ± SD of the three experiments. ns, not significant; *P < 0.05; **P < 0.01; ***P < 0.001; Student’s *t* test

In order to gain an understanding of the biological consequences underlying the dysfunction of ACAA1 enzyme at the molecular and cellular levels, we performed cellular assays using the U251 glioma cell line and the human microglia (HM) cell line overexpressing ACAA1 WT and p.N299S, respectively. The U251 cells were of astrocyte origin and engineered to consistently express mutant APP p.K670N/M671L (U251-APP) and produced Aβ under doxorubicin induction in our previous studies.^36,37^ RNA sequencing (RNA-seq) analyses were performed for the HM and U251-APP cells overexpressed with empty vector, ACAA1 WT, and ACAA1 p.N299S, respectively. We observed a clear distinction between the ACAA1 p.N299S and ACAA1 WT groups and between the ACAA1 p.N299S and the empty vector groups for both HM and U251-APP cells based on the principal component analysis (Supplementaty Fig. S2a). The heatmap of the dysregulated genes in both cell lines also showed a significant difference between the ACAA1 p.N299S and ACAA1 WT groups (Supplementaty Fig. S2b), indicating that the mutant p.N299S has an effect on the gene expression pattern. We identified 1219 differentially expressed genes (DEGs; *P*_adjust_ < 0.05) between the ACAA1 p.N299S and ACAA1 WT groups that were shared by both the HM and U251-APP cells, of which 734 genes were upregulated and 485 were downregulated (Supplementaty Fig. S2c). According to Kyoto Encyclopedia of Genes and Genomes (KEGG) pathway^38^ and Gene Ontology (GO) biological processes enrichment analyses,^39^ these DEGs were enriched in processes involved in lysosomal activity, cellular senescence, axon guidance, synaptic plasticity, fatty acid oxidation, and cognition (FDR [false discovery rate] <0.05, Fig. 2a and Supplementaty Table S3). Gene Set Enrichment Analysis (GSEA)^40^ indicated that these DEGs were strongly enriched in the GO terms “neurogenesis,” “neuron development,” and “neuron differentiation” (FDR < 1.00 × 10^−6^, Figs. 2b and Supplementaty Fig. S2d). KEGG pathway enrichment analysis of the U251-APP cells also showed that dysregulated genes in the ACAA1 p.N299S group were significantly enriched in the AD pathway (hsa05010, FDR = 2.26 × 10^−18^; Supplementaty Table S3), consistent with the background introduction of mutant APP p.K670N/M671L in this cell line.^36,37^

The DEGs between ACAA1 p.N299S and ACAA1 WT were significantly enriched (*P* < 0.05, Fisher’s exact test) in a co-expression network (*P*_adjust_ < 0.0001) (Fig. 2c) that was found to be abnormally regulated in brain tissues of AD patients,^41^ in which *ACAA1* was located in a central position of the network. A group of genes associated with late-onset AD implicated in lipid metabolism (*APOE* [apolipoprotein E]),^42,43^ immune response (*APOE*, *AGT* [angiotensinogen]),^41^ and the lysosome pathway (*CTSF* [cathepsin F], *LAMP1* [lysosomal associated membrane protein 1])^44^ were involved in this network. *COMT* (catechol-*O*-methyltransferase), a previously reported AD gene,^45^ was also dysregulated by ACAA1 p.N299S (Fig. 2c).

The lysosome-related pathologies, together with neuron loss and Aβ plaque deposition, have been shown to be accentuated in EOFAD due to mutations in PSEN1.^46^ We tested the protein levels of lysosomal markers LAMP1 and LAMP2A (lysosomal-associated membrane protein 2), autophagy markers MAP1LC3/LC3 (microtubule-associated protein 1 light chain 3) and SQSTM1 (sequestosome 1), and synaptic markers DLG4/PSD95 (Discs large MAGUK scaffold protein 4) and SYP (synaptophysin) in cells overexpressing ACAA1 p.N299S compared to those of cells overexpressing ACAA1 WT. We found a significant decrease in the protein levels of LAMP1, LAMP2A, PSD95, and SYP and a significant increase in the levels of LC3-II:LC3-I ratio and SQSTM1 in cells overexpressing ACAA1 p.N299S, suggesting potential dysfunction of synaptic transmission and lysosomal function in HM cells (Fig. 2d) and U251-APP cells (Fig. 2e). To characterize the roles of *ACAA1* in lysosomal and synaptic function, we generated ACAA1-deficient HM and U251-APP cells (ACAA1-KO) using the CRISPR/Cas9-mediated genome editing method (Supplementaty Fig. S3a, b). Consistent with the overexpression effect of ACAA1 p.N299S (Fig. 2d, e), knockout (KO) of ACAA1 significantly impaired the lysosomal and synaptic protein expression, as characterized by the reduced protein levels of PSD95, SYP, LAMP1, LAMP2A, CTSD (cathepsin D), CTSB (cathepsin B), and TFEB (transcription factor EB) and the increased LC3-II:LC3-I ratio and SQSTM1 protein level in both ACAA1-deficient U251-APP (Supplementaty Fig. S3c) and HM cells (Supplementaty Fig. S3d). We repeated the above results in a neuronal cell line SH-SY5Y cells by using small interfering RNA (siRNA) knockdown assays. Three siRNAs were designed for *ACAA1* and siRNA-1 (25 nM) was found to have the best inhibitory effect (Supplementaty Fig. S4) and was used in the following assays. We confirmed that both ACAA1 knockdown and ACAA1 p.N299S overexpression impaired lysosomal and synaptic protein expression in SH-SY5Y cells (Supplementaty Fig. S5). These data suggested that endogenous *ACAA1* was critically involved in lysosomal and synaptic function, and ACAA1 p.N299S might impair lysosomal function and disturb synaptic function.

### Virus-mediated overexpression of ACAA1 p.N299S exacerbated cognitive decline in APP/PSΔE9 mice

We investigated whether ACAA1 p.N299S would play a vital role in AD pathogenesis by using the adeno-associated virus (AAV php.eb) vector-mediated overexpression of ACAA1 WT and p.N299S in APPswe/PSEN1dE9 (APP/PS1ΔE9) mice. The APP/PS1ΔE9 mouse line contains human APP pathogenic mutations (Swedish mutations K595N/M596L) and mutated PSEN1/PS1 (presenilin 1) lacking exon 9.^47^ The AAV overexpression system is safe for gene delivery to the brains of rodents, monkeys, and humans.^48–50^ The AAV php.eb vectors expressing either green fluorescent protein (GFP) (AAV-Vector) or codon-optimized GFP-tagged human ACAA1 WT (AAV-ACAA1 WT) and ACAA1 p.N299S (AAV-ACAA1 N299S) under the control of the cytomegalovirus (CMV) promoter (Supplementaty Fig. S6a) were bilaterally injected into the hippocampus region of presymptomatic APP/PS1ΔE9 mice and WT littermates (3-month-old), respectively. As the spatial memory impairments and progressive Aβ plaque deposition in APP/PS1ΔE9 mice occurred at about at 15–17 weeks^51^ and 5–6 months of age,^47,52^ respectively, we assessed the effects of ACAA1 WT and ACAA1 p.N299S overexpression on the behavioral performance in APP/PS1ΔE9 mice and WT littermates 5 months later after AAV delivery.

**Fig. 3:**
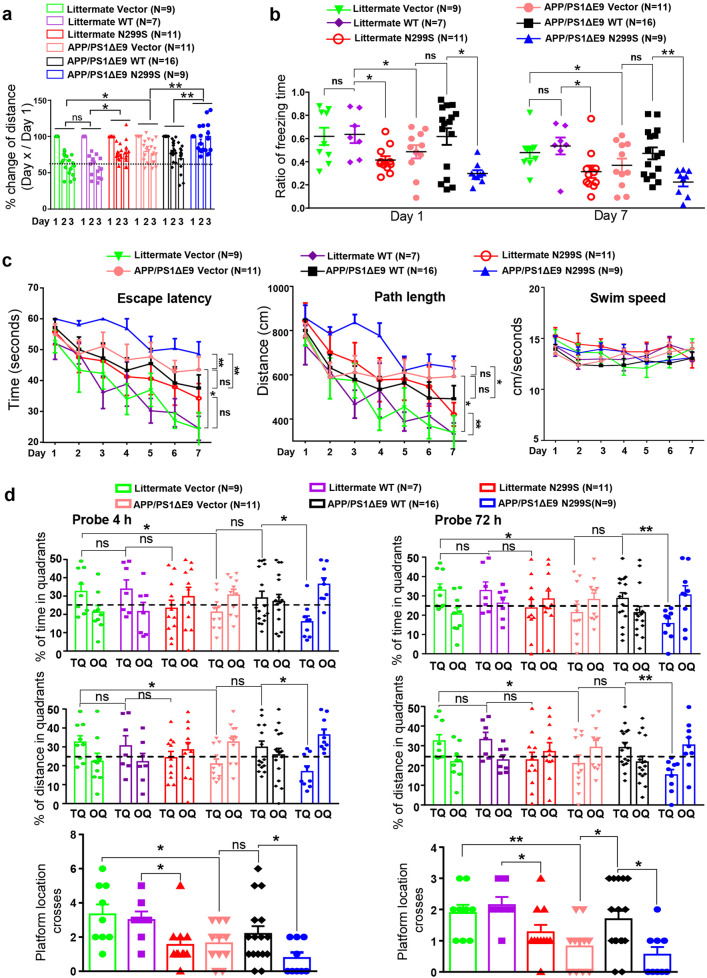
ACAA1 p.N299S aggravated memory impairments in APP/PS1ΔE9 mice. **a** Impaired habituation in the exploratory open field for APP/PS1 mice (8-month-old) and wild-type littermates with delivery of AAV-ACAA1 N299S versus AAV-ACAA1 WT or empty vector. The changes of distance traveled on days 2 and 3 were normalized to the distance traveled on day 1 of training. Data are mean ± SEM. ns, not significant; *P < 0.05; **P < 0.01, two-way repeated-measures ANOVA. **b** ACAA1 p.N299S accelerated memory retrieval impairment of APP/PS1 and WT mice in fear conditioning tests. Shown data are the percentages of freezing time after 1 day (left) and 7 days (right) of electric shocks. Data are mean ± SEM. ns, not significant; *P < 0.05; **P < 0.01, one-way ANOVA with the Tukey’s post hoc test. **c**, **d** Morris water maze tests of APP/PS1ΔE9 mice or WT littermates with delivery of AAV empty vector or AAV-mediated expression of ACAA1 WT and ACAA1 N299S. The APP/PS1ΔE9 mice or WT littermates with AAV-Vector, AAV-ACAA1 WT, or AAV-ACAA1 N299S injection showed differences in escape latency, path length, and swim speed during learning session (**c**) and in probe trial performance at 4 h (short-term memory; left panel) and at 72 h (right panel) (**d**). TQ target quadrant (percentage of time and percentage of distance in the target quadrant), OQ opposite quadrant. Bars represent mean ± SEM. *P < 0.05; **P < 0.01; one-way ANOVA with the Tukey’s post hoc test

In an exploratory open field test, the WT littermates injected with AAV-Vector and AAV-ACAA1 WT showed similar levels of habituation ability in detecting novel environments during the 3-day training course, whereas AAV-ACAA1 N299S-injected WT littermates showed a lower habituation ability compared to those injected with AAV-ACAA1 WT (Fig. 3a). The APP/PS1ΔE9 mice had an overall lower habituation ability to the testing environment compared to the WT littermates for each treatment. Consistent with the pattern in WT littermates, APP/PS1ΔE9 mice injected with AAV-Vector and AAV-ACAA1 WT showed the same habituation pattern, but the AAV-ACAA1 N299S-injected APP/PS1ΔE9 mice exhibited a significantly impaired habituation ability to the novel environment compared to the APP/PS1ΔE9 mice injected with AAV-Vector or AAV-ACAA1 WT (Fig. 3a).

The memory formation and retrieval abilities of the APP/PS1ΔE9 mice injected with AAV-ACAA1 WT and AAV-ACAA1 N299S were assessed by using the contextual fear conditioning test, in which their freezing responses were quantified at 1 and 7 days after the administration of an electric shock.^53^ Compared to the WT littermates, the APP/PS1ΔE9 mice had a reduction of freezing behavior at both 1 and 7 days after the electric shock, suggesting an impaired contextual retrieval of fear memory (Fig. 3b). The APP/PS1ΔE9 mice or WT littermates injected with AAV-ACAA1 WT had no significant alterations of freezing performance at both 1 and 7 days after the electric shock compared to the respective group injected with AAV-Vector group (Fig. 3b), although there is a tendency for better effect of AAV-ACAA1 WT in APP/PS1ΔE9 mice. Delivery of AAV-ACAA1 N299S aggravated the impaired freezing responses in the APP/PS1ΔE9 mice and WT littermates compared to the respective group injected with AAV-ACAA1 WT at both testing time points (Fig. 3b).

To look further at the subsequent consequences of AAV-ACAA1 WT or AAV-ACAA1 N299S delivery on spatial learning and memory, we carried out a Morris water maze place navigation task with these animals (Fig. 3c, d). Comparable swimming speed was found in all the groups of the APP/PS1ΔE9 mice and their WT littermates regardless of the injection with AAV-ACAA1 WT or AAV-ACAA1 N299S, which indicated no significant neuromotor differences among the groups (Fig. 3c). Although all APP/PS1ΔE9 mice and WT littermates managed the task after a 7-day training period, a significantly impaired learning ability was observed in APP/PS1ΔE9 mice compared to their WT littermates (Fig. 3c), consistent with our previous study^54^ and others.^55^ We observed a significantly impaired learning in the APP/PS1ΔE9 mice given the AAV-ACAA1 N299S compared to these injected AAV-ACAA1 WT or AAV-Vector (Fig. 3c), and a similar pattern was observed in the WT littermate groups. A 4-h and a 72-h probe trials after the last training session were further performed to evaluate impairment of short- and long-term memory for spatial reference, respectively. A greater memory loss was found in APP/PS1ΔE9 mice compared to WT littermates injected with AAV-Vector (using percentage of time, percentage of distance in the target quadrant, and the number of platform location crosses as readouts; Fig. 3d). The WT littermates treated with AAV-Vector, AAV-ACAA1 WT, or AAV-ACAA1 N299S exhibited similar preferences for both tests, except for the AAV-ACAA1 N299S group that had fewer platform location crosses compared to the AAV-ACAA1 WT group (Fig. 3d). Intriguingly, AAV-ACAA1 N299S-treated APP/PS1ΔE9 mice had inferior preference for the target quadrant relative to the APP/PS1ΔE9 AAV-ACAA1 WT group, suggesting that memory formation deficits in APP/PS1ΔE9 mice were exacerbated by ACAA1 p.N299S overexpression. The number of platform location crosses was significantly increased in the AAV-ACAA1 WT group compared to the AAV-Vector group in APP/PS1ΔE9 mice in the 72-h probe trial, indicating potentially beneficial effect of delay in the development of AD in this murine model (Fig. 3d). Taken together, these behavioral tests suggested a detrimental effect of ACAA1 p.N299S during the memory consolidation phase.

### Overexpression of ACAA1 p.N299S accelerated AD pathology in APP/PS1ΔE9 mice

**Fig. 4:**
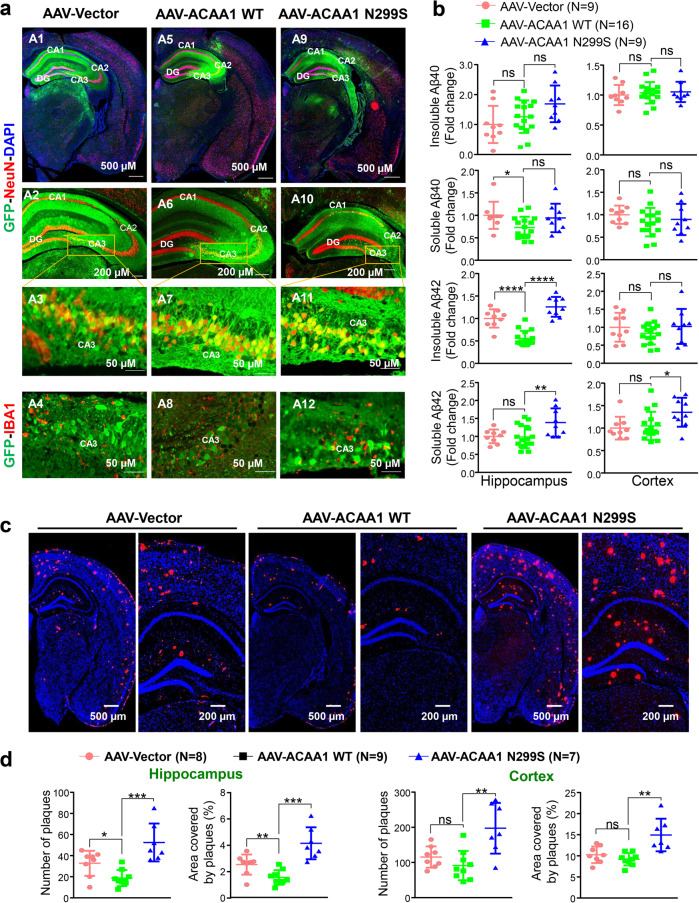
Overexpression of ACAA1 p.N299S via adeno-associated virus delivery exacerbated amyloid-β (Aβ) pathology. APP/PS1∆E9 mice and WT littermates were injected with AAV-Vector, AAV-ACAA1 WT, or AAV-ACAA1 N299S at 3 months of age and sacrificed at 9 months of age. **a** Fluorescent signals in the brain section of a 9-month-old mouse receiving AAV-Vector, AAV-ACAA1 WT, or AAV-ACAA1 N299S. **b** Quantification of soluble and insoluble Aβ40 and Aβ42 in the hippocampus and cortex tissues of mice in **a** by ELISA. **c**, **d** Representative microphotographs of hippocampal sections stained with an 4G8-specific Aβ antibody (**c**), and quantitative analysis of the number of Aβ plaques shown by 4G8 immunoreactivity in hippocampal and cortex tissues in APP/PS1ΔE9 mice (**d**). Bars represent mean ± SD. ns, not significant; *P < 0.05; **P < 0.01; ***P < 0.001; ****P < 0.0001; Student’s *t* test

We examined the AD pathological changes in the brain tissues of APP/PS1ΔE9 mice with delivery of ACAA1 WT and ACAA1 p.N299S at 6 months after AAV injection. Consistent with the injection location, immunohistochemistry using a GFP-tag-specific antibody showed a widespread overexpression of ACAA1 WT and ACAA1 p.N299S in the hippocampus tissues, with prominent expression of ACAA1-GFP in the neurons of the CA1, CA2, and CA3 regions, especially within the CA3 subfield (Fig. 4a). As ACAA1 expression was driven by the CMV promoter, ACAA1-GFP expression had no cell-specific pattern and was detectable in both neuronal cells (positive immunostaining against NeuN [RBFOX3, RNA binding fox-1 homolog 3] and GFP) and microglia (positive immunostaining against IBA1 [AIF1, allograft inflammatory factor 1] and GFP) (Fig. 4a).

Measurement of soluble and insoluble Aβ levels by using enzyme-linked immunosorbent assay (ELISA) showed that soluble Aβ42/Aβ1-42 species, which are synaptotoxic in AD,^56^ were significantly increased in both the hippocampus and cortex tissues of APP/PS1ΔE9 mice with overexpression of ACAA1 p.N299S relative to the ACAA1 WT group (Fig. 4b). The levels of insoluble Aβ42 were also increased in the hippocampus, but not in the cortex tissues of APP/PS1ΔE9 mice injected with AAV-ACAA1 p.N299S relative to AAV-ACAA1 WT (Fig. 4b). However, both soluble and insoluble Aβ40/Aβ1-40 species were not significantly changed in the hippocampus and cortex tissues of the ACAA1 p.N299S group compared to the ACAA1 WT group. Overexpression of ACAA1 WT could reduce the level of soluble Aβ40 compared to the empty vector group in the hippocampus tissues of APP/PS1ΔE9 mice (Fig. 4b).

We assessed the effect of ACAA1 p.N299S overexpression on amyloid deposition by using Aβ (antibody 4G8) immunostaining and quantified the area covered by Aβ plaques in both the hippocampus and cortex tissues of APP/PS1ΔE9 mice. A significantly increased burden of 4G8-labeled Aβ plaques was observed in hippocampus and cortex tissues of APP/PS1ΔE9 mice after delivery of ACAA1 p.N299S compared to those groups injected with the empty vector and ACAA1 WT (Fig. 4c, d). Similar to the ELISA results for soluble Aβ40 and insoluble Aβ42, delivery of ACAA1 WT reduced the number of Aβ plaques compared to the empty vector in the hippocampus tissue of APP/PS1ΔE9 mice. Immunohistochemical staining analysis also showed a significant increase of 4G8-labeled Aβ plaque burden in coronal brain sections of APP/PS1ΔE9 mice after AAV-ACAA1 N299S injection compared to those with AAV-ACAA1 WT injection (Supplementaty Fig. S6b, c). Together, all these findings demonstrated that overexpression of ACAA1 p.N299S accelerated Aβ pathology in APP/PS1ΔE9 mice.

### Overexpression of ACAA1 p.N299S caused neuron loss in the hippocampal CA3 region and disturbed synaptic function

**Fig. 5:**
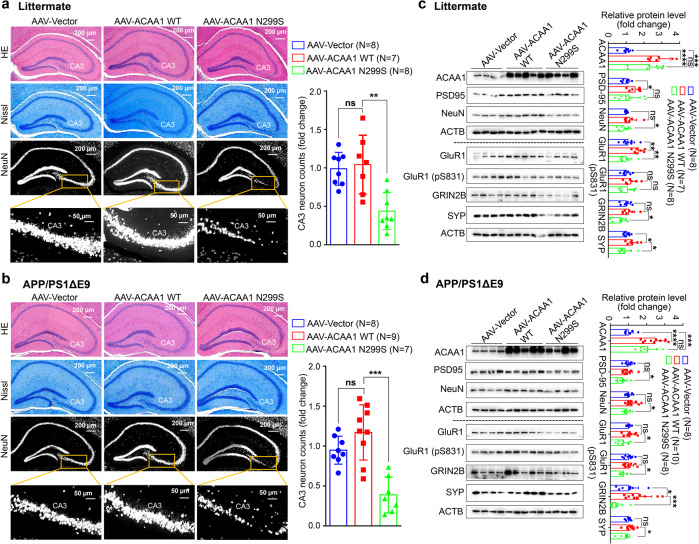
Overexpression of ACAA1 p.N299S in APP/PS1ΔE9 mice and WT littermates induced neuronal losses. **a**, **b** Neuronal loss in the CA3 region of hippocampus in WT littermates (**a**) and APP/PS1ΔE9 mice (**b**) after delivery of the AAV-Vector, AAV-ACAA1 WT, and AAV-ACAA1 N299S. Higher neuronal density, marked by NeuN antibody, was noted in mice overexpressing ACAA1 p.N299S, suggesting a potential decrease of neurogenesis or an increase of neuron loss. Immunostaining of brain sections was performed using NeuN antibody. Shown results are representative hematoxylin and eosin (H&E) staining (top), Nissl staining (middle), and NeuN staining (bottom) of mouse hippocampus tissues in each group. **c**, **d** Decreased protein levels of structural plasticity markers SYP, PSD95, NeuN, GluR1, GluR1 (pS831), and GRIN2B in the hippocampus tissues of WT littermates (**c**) and APP/PS1ΔE9 (**d**) mice with AAV-mediated expression of ACAA1 and its mutant. Bars represent mean ± SD. ns, not significant; *P < 0.05; **P < 0.01; ***P < 0.001; ****P < 0.0001; Student’s *t* test

We examined hippocampal morphology of neuronal cells using hematoxylin and eosin (H&E) staining and Nissl staining to discern the deleterious effect of ACAA1 p.N299S. The number of neurons in the hippocampal CA3 region was significantly decreased in the WT littermates and APP/PS1ΔE9 mice with delivery of AAV-ACAA1 N299S compared to the respective group with AAV-ACAA1 WT. H&E staining and Nissl staining of brain sections also showed a significant shrinkage of the hippocampus CA3 region in animals with AAV-ACAA1 N299S injection (Fig. 5a, b). The number of NeuN (neuron marker)-positive neuronal cells in the hippocampus CA3 region of WT littermates and APP/PS1ΔE9 mice injected with AAV-ACAA1 N299S was also significantly decreased compared to the respective group with AAV-ACAA1 WT (Fig. 5a, b). These results indicated that ACAA1 p.N299S overexpression induced neuronal loss.

Consistent with the decreased levels of structural neuroplasticity markers PSD-95 and SYP in HM cells and U251-APP cells with overexpression of ACAA1 p.N299S relative to cells overexpressing ACAA1 WT (Fig. 2d, e), the levels of these proteins were significantly decreased in hippocampus tissues of WT littermates and APP/PS1ΔE9 mice after the delivery of AAV-ACAA1 N299S compared to AAV-ACAA1 WT (Fig. 5c, d). This effect was also consistent with the effect of ACAA1 KO in HM cells and U251-APP cells (Supplementaty Fig. S3), indicating ACAA1 p.N299S as a loss-of-function mutation. Moreover, the levels of these proteins that are actively involved in synaptic plasticity, such as NeuN, GluR1, GluR1 (pS831), and GRIN2B, were also decreased in the hippocampus tissues of WT littermates and APP/PS1ΔE9 mice after AAV-ACAA1 p.N299S delivery compared to the respective group injected with AAV-ACAA1 WT (Fig. 5c, d).

### Acceleration of Aβ pathology by ACAA1 p.N299S was mediated by impaired lysosomal function

**Fig. 6:**
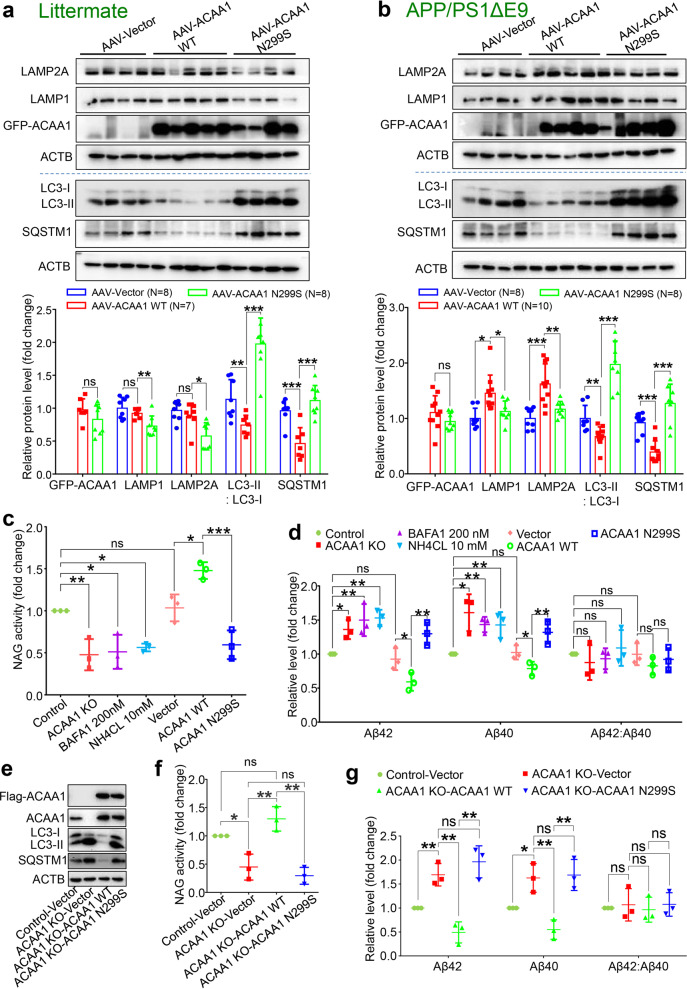
Effect of ACAA1 p.N299S on Aβ pathology was mediated by lysosomal dysfunction. **a**, **b** Western blot analysis of lysosomal proteins LAMP1 and LAMP2A and autophagy markers LC3-II:LC3-I ratio and SQSTM1 in the hippocampus tissues of WT littermates (**a**) and APP/PS1∆E9 (**b**) injected with AAV-ACAAA WT or AAV-ACAA1 N299S. Bars represent mean ± SD. ns, not significant; *P < 0.05; **P < 0.01; ***P < 0.001; Student’s *t* test. (**c**) *ACAA1* knockout (KO) or overexpression of ACAA1 p.N299S in U251-APP cells decreased NAG activity. Cells were treated with BAFA1 (200 nM), NH4CL (10 mM), or without treatment (Control) or were transfected with empty vector (Vector) and expression vector of ACAA1 WT, or ACAA1 p.N299S. **d** Levels of extracellular Aβ42, Aβ40, and Aβ42:Aβ40 ratio in the culture supernatants of U251-APP cells and U251-APP *ACAA1* KO cells. **e**–**g** Overexpression of ACAA1 WT, but not ACAA1 p.N299S, in U251-APP ACAA1 KO cells had a rescuing effect on the altered protein levels of autophagy markers (**e**), lysosomal markers (**f**), and the levels of extracellular Aβ42 and Aβ40 in the culture supernatant (**g**). The U251-APP cells without ACAA1 knockout was used as a control, and cells were transfected with empty vector (Vector) and expression vector of ACAA1 WT or ACAA1 p.N299S. Data in **e**–**g** were based on three independent experiments. Bars represent mean ± SD. ns, not significant; *P < 0.05; **P < 0.01; ***P < 0.001; Student’s *t* test

Dysfunction of the lysosome and autophagy has been actively involved in neurodegeneration.^46,57–59^ Overexpression of ACAA1 p.N299S or ACAA1 KO in HM cells and U251-APP cells decreased lysosomal marker proteins LAMP1 and LAMP2A and increased LC3-II:LC3-I ratio and SQSTM1 protein level (Supplementaty Figs. 2d, e and S3c, d), and this observation could be validated in SH-SY5Y cells (Supplementaty Fig. S5), suggesting reduced lysosomal activity caused by ACAA1 p.N299S or ACAA1 KO. We next tested whether the increased level of Aβ in APP/PS1ΔE9 mice with delivery of ACAA1 p.N299S (Figs. 4 and Supplementaty Fig. S6b, c) was associated with lysosomal dysfunction. We quantified the LAMP1 and LAMP2A protein levels in hippocampus tissues of APP/PS1ΔE9 mice and WT littermates injected with AAV-ACAA1 WT and AAV-ACAA1 N299S. Overexpression of ACAA1 p.N299S caused a significant decrease of LAMP1 and LAMP2A in the hippocampus tissues compared to overexpression of ACAA1 WT in APP/PS1ΔE9 mice and WT littermates, respectively (Fig. 6a, b). Similarly, overexpression of ACAA1 p.N299S led to an increased LC3-II:LC3-I ratio and SQSTM1 protein level in the hippocampus tissues of APP/PS1ΔE9 mice and WT littermates compared to the respective group with ACAA1 WT overexpression (Fig. 6a, b).

To test whether the increased Aβ accumulation upon ACAA1 p.N299S overexpression was caused by lysosomal dysfunction and autolysosome defect, we used BAFA1 and NH4CL, inhibitors of the vacuolar (V)-type ATPase that results in blockage of autophagosome–lysosome fusion and accumulation of LC3B,^60,61^ as the positive controls to treat U251-APP cells and determined the level of Aβ in cell culture supernatant. Lysosomal function was inhibited in U251-APP cells (Fig. 6c) and HM cells (Supplementaty Fig. S7a) by BAFA1 and NH4CL treatments, as well as in cells overexpressing ACAA1 p.N299S or ACAA1 KO, as indicated by the decreased lysosomal protease activities according to the β-*N*-acetylglucosaminidase (NAG) assays. This dysfunction effect was accompanied by increased extracellular Aβ40 and Aβ42 levels in comparison with untreated cells (Fig. 6d). Treatment of BAFA1 (200 nM) and NH4CL (10 mM) alone raised the extracellular Aβ40 and Aβ42 levels compared to untreated cells (Fig. 6d), and this effect was comparable to the cells overexpressing ACAA1 p.N299S or with ACAA1 KO. Concordantly, overexpression of ACAA1 p.N299S inhibited lysosomal function and autophagosome–lysosome fusion in U251-APP cells (Supplementaty Fig. S7b) and HM cells (Supplementaty Fig. S7c), and this effect was similar to that of ACAA1 KO or BAFA1 and NH4CL treatments (Supplementaty Fig. S7b, c).

We performed rescue experiments using the U251-APP and HM ACAA1 KO cells. Overexpression of ACAA1 WT rescued the altered LC3-II:LC3-I ratio and SQSTM1 protein level (Figs. 6e and Supplementaty Fig. S7d) and lysosomal activity (Figs. 6f and Supplementaty Fig. S7e) in ACAA1 KO cells, whereas ACAA1 p.N299S overexpression had no such an effect. Accordingly, overexpression of ACAA1 WT, but not ACAA1 p.N299S, ablated the increased extracellular Aβ40 and Aβ42 levels in U251-APP ACAA1 KO cells (Fig. 6g). As the Aβ42:Aβ40 ratio was not significantly changed in these conditions, we measured the protein levels of BACE1, PSEN1, PSEN2, and PEN2 in U251-APP cells with or without the respective treatment and transfection. We found no significant alterations of BACE1, PSEN1, PSEN2, and PEN2 protein levels between cells with or without ACAA1 KO. Similarly, no difference of these protein levels was found between cells with or without chemical treatments or between cells with overexpression of ACAA1 WT and p.N299S (Supplementaty Fig. S7f). This result suggested that the increased levels of Aβ40 and Aβ42 in cells with ACAA1 KO or ACAA1 p.N299S overexpression might be caused by the lysosomal dysfunction and impaired autophagosome–lysosome fusion. Together, these findings demonstrated that ACAA1 p.N299S accelerates Aβ burden.

### Overexpression of ACAA1 p.N299S affected excitatory synaptic transmission

**Fig. 7:**
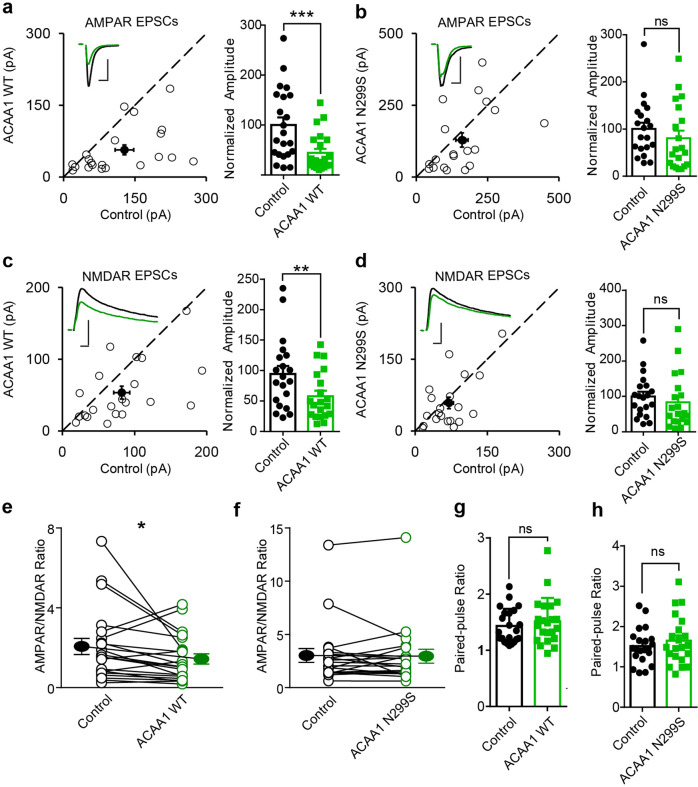
ACAA1 p.N299S disturbs its physiological regulation of excitatory synaptic transmission. **a**–**d** Rat hippocampal slice cultures were biolistically transfected with expression vector of ACAA1 WT or ACAA1 p.N299S. Simultaneous dual whole-cell recordings were performed in a transfected CA1 pyramidal neuron (green trace) and a neighboring wild-type one (black trace). The evoked AMPA (**a**, **b**) and NMDA (**c**, **d**) EPSCs were measured, and open and filled circles represent amplitudes for single pairs and mean ± SEM, respectively. Sample current traces from control (black) and experimental (green) cells are shown as insets. Bar graphs show normalized EPSC amplitudes (mean ± SEM) of −70 mV (**a**, ***P < 0.001; **b**, *P* > 0.05) and +40 mV (**c**, **P < 0.005; **d**, *P* > 0.05) presented in scatter plots. The scale bars for representative EPSC traces are 100 pA/25 ms (**a**) and 50 pA/25 ms (**b**–**d**). **e**, **f** Difference of AMPA/NMDA ratios recorded from neurons overexpressing ACAA1 WT (*P < 0.05) or ACAA1 p.N299S (*P* > 0.05) compared to the respective wild-type (Control) ones. **g**, **h** No change in paired-pulse ratio of the second EPSC over the first EPSC from neurons overexpressing ACAA1 WT (*P* > 0.05) or ACAA1 p.N299S (*P* > 0.05) relative to the control neurons. All the statistical differences are estimated relative to the respective control neurons, with a two-tailed Wilcoxon signed-rank sum test

As ACAA1 p.N299S overexpression reduced the structural neural protein levels and was associated with Aβ accumulation, we determined whether this mutation directly alters excitatory neuron synaptic transmission. We used rat hippocampal CA1 pyramidal neurons and dual whole-cell recordings described in our previous studies,^21,62^ to investigate the electrophysiological effect of ACAA1 WT and p.N299S. Compared to the control neurons, overexpression of ACAA1 WT decreased the AMPA receptor (AMPAR)-mediated synaptic transmission (Fig. 7a), whereas ACAA1 p.N299S had no such an effect (Fig. 7b). Similarly, the *N*-methyl-d-aspartate receptor (NMDAR)-mediated synaptic transmission was inhibited significantly by overexpression of ACAA1 WT, but overexpression of ACAA1 p.N299S did not change NMDAR-evoked excitatory postsynaptic currents (EPSCs) (Fig. 7c, d). Overexpression of ACAA1 WT, but not ACAA1 p.N299S, decreased the ratio of AMPAR and NMADR-mediated EPSCs when compared to neighboring wild-type neurons in respective assays (Fig. 7e, f). Nonetheless, both ACAA1 WT and p.N299S overexpression did not affect the paired-pulse ratio (Fig. 7g, h), which reflects presynaptic release probability. Collectively, these results suggested that ACAA1 WT had an active role in modulating the excitatory synaptic transmission mediated by both AMPAR and NMDAR in neurons, whereas ACAA1 p.N299S impaired this regulation possibly via altered levels of proteins involved in synaptic functions (Fig. 5) and/or a gain of toxic function. Focused experiments should be performed to test this hypothesis and to elucidate the underlying mechanism.

## Discussion

**Fig. 8:**
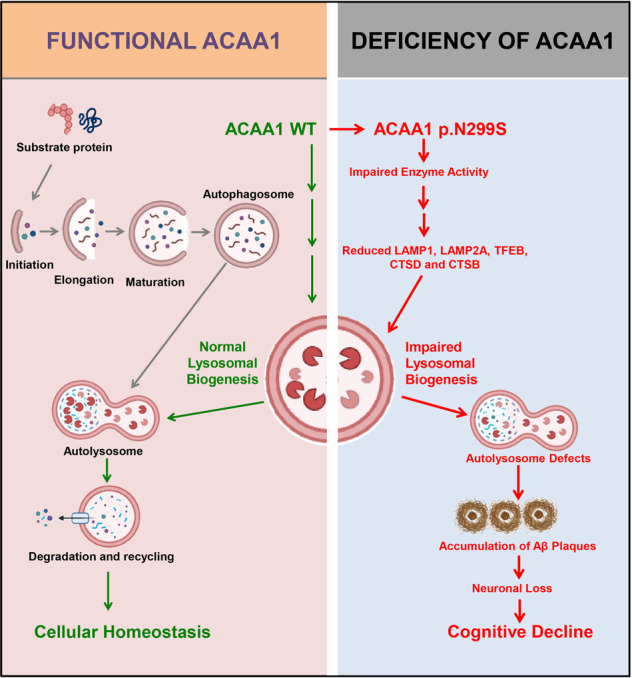
A proposed role of the ACAA1 p.N299S-mediated lysosomal dysfunction and impaired autophagy in the development of AD. ACAA1 p.N299S has an impaired enzymatic activity, affects autophagy and lysosomal function, subsequently contributes to the aggravation of the Aβ pathology and neuronal loss, and finally causes cognitive impairment and other AD-related symptoms

Accumulating evidence has shown that AD has a genetic basis, with contributions from multiple causal and risk genes.^13,14,17,22^ Molecular characterization of these genes provides insights into understanding AD pathobiology and developing drug therapy. In this study, we identified an association of the missense variant ACAA1 p.N299S with AD in EOFAD patients (Fig. 1a and Supplementaty Table S2), which showed a population-specific pattern. We have further provided in vitro and in vivo data showing that this ACAA1 variant facilitates Aβ pathology and exacerbates cognitive decline by impairing lysosomal and synaptic function, adding this gene to the current list of AD risk and causal genes.^13,14,17,22,41^ ACAA1 p.N299S is a loss-of-function mutant, as it decreases enzymatic activity (Fig. 1b) and causes a catastrophic cascade with the involvement of disturbed global gene expression pattern and impaired lysosomal, autolysosomal, and synaptic functions (Fig. 2). Using AAV-mediated overexpression of ACAA1 p.N299S in APP/PS1ΔE9 mice exacerbated cognition decline (Fig. 3), accelerated Aβ pathology (Figs. 4, 6 and S6), and impaired synaptic protein expression and neuronal loss in the hippocampus CA3 region (Fig. 5). Moreover, overexpression of ACAA1 p.N299S disturbed the excitatory synaptic transmission in rat hippocampus Purkinje neurons as compared to ACAA1 WT (Fig. 7). All these results were compatible with the impaired lysosomal function and autophagosome–lysosome fusion that were caused by the ACAA1 p.N299S (Fig. 8).

The involvement of ACAA1 in lipid metabolism as part of the development of AD can be explained and has many important implications. First, previous studies had shown that lipids are crucial for maintaining neuronal development, synaptic plasticity, and function.^63,64^ Abnormal lipid metabolism is actively involved in the development of neurodegenerative diseases, including AD.^65,66^ Moreover, dysfunction of VLCFA β-oxidation in peroxisomes is a common feature of some neurodegenerative diseases,^67,68^ although the molecular underpinning underlying the neuron loss vary widely.^68,69^ In particular, peroxisomal function declines with age and is linked to AD,^69,70^ with an increased levels of VLCFAs in the AD brains,^69^ suggesting a possible defect in peroxisomal β-oxidation during the development of AD. Second, numerous studies have demonstrated that loss of peroxisomal proteins and enzymes constitutes one of the reasons for severe neuronal defects,^71,72^ as peroxisomes are common in these neuronal cells, such as neurons, astrocytes, oligodendrocytes, microglia, and Schwann cells.^73,74^ Indeed, mice lacking the peroxisomal proteins PEX5^75^ and PEX10^76^ display severe neurological defects. Loss-of-function mutations in ACOX1 (acyl-CoA oxidase 1), the first and rate-limiting enzyme of the VLCFA β-oxidation signaling pathway in peroxisomes, impair synaptic transmission and cause glial and axonal loss.^77^ Similarly, mice deficient for MFP2, a VLCFA metabolizing enzyme upstream of ACAA1, also exhibit a severe loss of axons.^78^ Impairment of axons has been frequently found in AD.^79,80^ Third, peroxisomal ACAA1 is the last and key enzyme in VLCFA β-oxidation and a main acetyl-CoA producer.^81,82^ The reduction of peroxisomal ACAA1 enzymatic activity decreases the rate of peroxisomal β-oxidation of palmitoyl-CoA.^83^ It has been reported that ACAA1 deficiency leads to pseudo-Zellweger syndrome,^83,84^ and emerging evidence has shown that AD and pseudo-Zellweger syndrome share a common risk of peroxisomal alterations.^69,85^ Therefore, our finding of ACAA1 p.N299S, a loss-of-function mutation in EOFAD patients, to be actively involved in Aβ pathology and cognitive decline in an AD murine model, further emphasizes the important role of peroxisomal protein dysfunction in neurodegeneration.

Dysfunctions in the lysosomal system are well-recognized early neuropathological features of AD, marked by prominent enlargement of endosomal compartments and lysosomal deficits.^59,86,87^ Lysosomes are major cellular degradative organelles, involved in turnover of molecular cargo from both autophagic and endocytic pathways,^87^ and in AD, disturbed lysosomal degradation is presumed to be of key importance in aberrant autophagic vacuole turnover.^86,88^ The lysosomal deficits in AD are thought to cause impaired autophagosome–lysosome fusion and disruption of substrate proteolysis within autolysosomes.^57–59,86^ Defective lysosomal proteolysis exacerbates Aβ pathology in mouse models of AD.^57,59,86^ We found that ACAA1 p.N299S impaired lysosomal function and cognitive function in both WT littermates and APP/PS1ΔE9 mice, suggesting that defective lysosomal production in neuropathology might be a common feature in neurodegenerative disease. The elimination of Aβ generated in the endocytic–autophagic pathways in neurons has a dependence on lysosomal degradation capacity.^57,89^ Consistent with this speculation, we found that ACAA1 p.N299S causes lysosomal inhibition, lead to an increased Aβ load, impaired synaptic function, and accelerated neuronal loss in AD. Importantly, AAV-ACAA1 p.N299S treatment leads to an impairment of spatial reference memory. The significant increment of soluble and insoluble Aβ42 and plaque burden, resulting in excessive Aβ neurotoxicity^54^ and lysosomal dysfunction in APP/PS1ΔE9 mice upon ACAA1 p.N299S overexpression, may account for the accelerated neuronal loss in APP/PS1ΔE9 mice with the administration of AAV-ACAA1 p.N299S. It should be mentioned that ACAA1 p.N299S also caused neuronal loss in WT littermates (Fig. 5a), which suggested that other factors caused by ACAA1 p.N299S were involved in this process. Further study should be carried out to confirm this speculation. We provided multiple lines of evidence to show that overexpression of ACAA1 p.N299S in APP/PS1ΔE9 mice significantly aggravated Aβ pathologies and Aβ-mediated neurodegeneration, supporting ACAA1 as a sensitizing factor for Aβ pathology and as a novel mechanism underlying the AD risk.

Neurotoxic Aβ oligomers can interact with and activate NMDA receptor^90,91^ and affect NMDA receptor signaling.^90,92,93^ Overactivation of NMDA receptors causes excitotoxicity and neuronal cell damage,^94^ whereas chronic NMDA receptor hyperactivity contributes to neuron loss in the development of AD.^95^ The Food and Drug Administration-approved drugs for treating AD, such as rivastigmine, galantamine, donepezil, memantine, memantine-donepezil combination, and tacrine, block glutamate NMDAR, inhibit acetylcholinesterase, or have a combination of both effects. We found that ACAA1 WT likely inhibited the excitatory synaptic transmission, whereas ACAA1 p.N299S impaired this regulation (Fig. 7). We speculated that ACAA1 p.N299S contributes to AD by disrupting the essential regulation of ACAA1 WT on the excitatory synaptic transmission via a currently unknown mechanism that awaits future focused assays. It would be rewarding to find whether ACAA1 can be a potential target in AD therapeutics, as overexpression of ACAA1 WT seems to have a beneficial role in reducing Aβ load and for maintaining presynaptic and postsynaptic integrity and function (Figs. 4 and 5).

This study had several limitations. First, the association of ACAA1 p.N299S with EOFAD had to be validated in independent populations and detailed analyses of clinical features of those AD patients carrying this mutation should be performed. Second, we found that p.N299S impairs the enzymatic activity of ACAA1, but the detailed mechanism as to how the reduced ACAA1 enzymatic activity is involved in the progress of AD and whether the lysosomal dysfunction induced by ACAA1 p.N299S has any cell-type specificities in the brain have not been sufficiently elucidated. Although the transcriptomic gene profiling in this study could offer some hints regarding the alterations of pathways and AD-centered network, the underlying pathway for lysosomal dysfunction caused by reduced peroxisomal ACAA1 activity has remained to be characterized. The altered protein levels of LC3-II/LC3-I and SQSTM1 in cells with or without BAFA1 and NH4CL treatment might be compatible with impaired autophagosome–lysosome fusion that was associated with abnormal Aβ42 deposition (Figs. 6 and Supplementaty Fig. S7). However, we did not perform a focused assay for autophagic flux in cells overexpressing ACAA1 p.N299S or with ACAA1 KO to characterize the potential role of autophagy due to technical reasons, especially considering the fact that we observed an increased LC3-II:LC3-I ratio and SQSTM1 protein level in both ACAA1-deficient cells or cells overexpressing the mutant. Third, although the AAV-mediated gene delivery might have some disadvantages as compared to knock-in animals for characterizing the consequence of ACAA1 p.N299S, we observed remarkable deficits in APP/PS1ΔE9 mice with AAV-ACAA1 p.N299S as compared to those with AAV-ACAA1 WT, such as cognition decline, Aβ accumulation, and neuronal loss (Figs. 3–6 and Supplementaty Fig. S6), which indicated the deleterious effect of ACAA1 p.N299S. Given the fact that neurons play key roles in AD and interactions between neurons and glial cells are actively involved in the pathobiology of AD,^65,96^ it would be worthwhile to detect the lysosomal and synaptic dysfunctions caused by ACAA1 p.N299S in neuronal cells and neuron–glia interaction models. Fourth, we did not analyze the role of tau in producing these effects mediated by ACAA1 p.N299S, which should be evaluated in future studies by incorporating tau into these models to determine whether ACAA1 p.N299S influences Aβ and tau during the progression of AD. Finally, we did not perform a drug screening using the ACAA1 as the target. Based on the current results that the WT littermates with AAV-ACAA1 N299S delivery already showed some deficits (Fig. 3), potential chemicals/drugs promoting lysosomal function and/or regulating the excitatory synaptic transmission (Fig. 7) could be expected to have an ameliorating effect on alleviating the deficits caused by ACAA1 p.N299S and other potentially pathogenic mutations in this gene. The complete picture of ACAA1 and its role in AD will be critical for answering the question as to whether this gene can be used as a druggable target for AD treatment.

In conclusion, we have provided multiple lines of supporting evidence showing peroxisomal ACAA1 contributing to abnormal lysosomal function in AD and for studying the causes of lysosomal dysfunction in AD patients. Overexpression of ACAA1 p.N299S has been shown to contribute to AD by disturbing its enzymatic activity, inhibiting the lysosome system, and aggravating the Aβ pathology and neuronal loss, which finally caused a cognitive impairment in a murine model of AD (Fig. 8). Our findings reveal a fundamental role of peroxisome-mediated lysosomal dysfunction in AD pathogenesis. It will be rewarding to perform a prospective drug study with ACAA1 as a valid druggable target for improving the pathological characteristics and cognitive impairment symptoms in AD patients with deficiency of ACAA1 enzyme activity and impaired VLCFA β-oxidation.

## Materials and methods

### Antibodies, chemicals, and vectors

Details of primary antibodies, secondary antibodies and chemicals used in this study are listed in Supplementaty Table S4. Vectors p3*Flag-CMV-14 (empty vector), p3*Flag-CMV-14-ACAA1 WT (PPL01228-2a) (ACAA1 WT), and p3*Flag-CMV-14-ACAA1 WT (PPL01228-2B) (ACAA1 N299S) were purchased from Public Protein/Plasmid library (Nanjing, Jiangsu).

### Human subjects

The members of the EOFAD family from Guizhou, Southwest China were enrolled in this study. The proband II:3 was diagnosed as having AD (age at onset [AAO] <57 years), and his mother I:2 was diagnosed as possibly having AD (AAO <60 years). Both patients had died before we could have a more focused clinical examination (Fig. 1a). The patients were initially diagnosed as having AD by at least two clinical psychiatrists according to the revised National Institute of Neurological and Communicative Disorders and Stroke and the Alzheimer’s Disease and Related Disorders Association criteria^97–99^ and the Diagnostic and Statistical Manual of Mental Disorders (Fourth Edition) as described in our previous studies.^36,37,100^ Samples were collected according to the Declaration of Helsinki, and the informed consents were obtained from the participants or supervisors of the patients. This study was approved by the Institutional Review Board of Kunming Institute of Zoology (KIZ), Chinese Academy of Sciences (CAS; approval numbers SMKX-20170103-01 and SMKX-SQ-20200414-074-02).

### WGS of the AD pedigree

Four individuals including the proband from the AD pedigree were subjected to WGS (Fig. 1a) and were processed using the same pipeline described in our previous study.^101^ Briefly, genomic DNAs were isolated from the peripheral blood by using AxyPrep Blood Genomic DNA Miniprep Kit (Axygen, USA). Deep WGS (~30×) was performed at the Novogene Corporation (Tianjin Novogene Technology Co., Ltd.) using Illumina HiSeq 4000 Platform (150 bp paired-ends reads). We removed low-quality bases of raw reads using Trimmomatic-0.32^102^ with the parameters “LEADING:3 TRAILING:3 SLIDINGWINDOW:4:15 MINLEN:36.” Quality-filtered reads were mapped to hg19 reference genome (https://www.ncbi.nlm.nih.gov/assembly/GCF_000001405.13/) by BWA version 0.79a.^103^ The aligned BAM files of each sample were sorted by genomic position using SortSam and merged into a single file using MergeSamFiles in picard-tools-1.107 (https://github.com/broadinstitute/picard). We used MarkDuplicates in picard-tools-1.107 to mark the duplicate reads for exclusion in the subsequent analyses. We used GATK version 2.8^104^ for calling single-nucleotide variants (SNVs) with the parameters as recommended (http://www.broadinstitute.org/gsa/wiki/index.php/Best_Practice_Variant_Detection_with_the_GATK_v3) and used the GATK UnifiedGenotyper (UG) to estimate genotype likelihoods in this family. To maximize sensitivity and correctness of SNV calling, we set the GATK UG with a Phred quality score > Q10 as a starting point, followed by filter using the GATK Variant Quality Score Recalibration (VQSR) to exclude spurious SNVs caused by sequencing and mapping artifacts. We annotated all variants according to RefSeq gene transcripts (accessed from the UCSC Genome Browser, http://genome.ucsc.edu) using our in-house script as previously described.^101^

We followed the same strategy in our previous studies^21,30^ to identify all rare (MAF ≤ 0.01 in the datasets of the 1000 Genomes Project^29,32^) and inherited loss-of-function (stop-gain or frameshift) and damaging missense variants (Supplementaty Tables S1 and S2) and used the dbNSFP database^105^ for functional prediction and annotation of SNVs. We also used the CADD score,^31^ a method integrating multiple annotations, to evaluate function potential of SNVs.

### Cell culture and western blotting

The U251 glioma, HM, and SH-SY5Y cells were introduced from Kunming Cell Bank, KIZ, CAS. The U251-APP cells with stably expression of mutant APP K670N/M671L produced Aβ under doxorubicin treatment were taken from our previous studies.^36,37^ Western blotting for target proteins were performed using the common approach as described in our previous studies^54,106^ and the respective antibodies listed in Supplementaty Table S4. The detailed information regarding cell culture, transfection, and western blotting can be found in the online Supplementary Materials and Methods.

### RNA-seq analysis and *ACAA1* co-expression network construction

We followed a similar pipeline and procedure in our previous studies^21,41^ to conduct the RNA-seq analysis and reconstruct the ACAA1 co-expression network. More details can be found in the online Supplementary Materials and Methods. We took the compiled expression matrix of 269 postmortem brain samples of AD patients from the AlzData database (www.alzdata.org),^41^ which contains reported microarray data of four AD brain tissues, including entorhinal cortex, hippocampus, temporal cortex, and frontal cortex,^41^ to discern the co-expression pattern of *ACAA1*. Spearman’s correlation coefficients and the Benjamini–Hochberg-adjusted *P* values (*P*_adjust_) were calculated using R package *psych*. The *ACAA1*-centered co-expression network was constructed using genes that are significantly correlated (*P*_adjust_ < 0.0001) with *ACAA1*. Fisher’s exact test was used to test the enrichment between DEG signatures of cells overexpressing ACAA1 p.N299S and ACAA1 WT or empty vector.

### Generation of *ACAA1* KO cell

We used the procedure described in our previous studies^107,108^ to KO *ACAA1* in the HM and U251-APP cells. Briefly, small guide RNAs (sgRNAs) (*ACAA1*-sgRNA-F: 5’-CACCGTGCCGAGAGAAGCTCGTCGG-3’/*ACAA1*-sgRNA-R: 5’-AAACCCGACGAGCTTCTCTCGGCAC-3’) targeting *ACAA1* were annealed and cloned into the pX330-T7 vector expressing mCherry. The HM and U251-APP cells were transfected with this vector carrying the sgRNAs by using Lipofectamine 3000 (Invitrogen, L3000008) for 48 h, then single cells expressing mCherry were sorted and cultured for 3 weeks for expansion. Genomic DNA was isolated from single HM and U251-APP cells with potential ACAA1 KO using AxyPrep Multisource Genomic DNA Miniprep Kit (Axygen, 26817KC1) and was amplified by using primer pair *ACAA1*-sgRNA-Fc: 5’-TGTGGTCGCTTTGTCTCCCT-3’/*ACAA1*-sgRNA-Rc: 5’-CTCCCATCTGACGAGAAATACCC-3’). Purified PCR products were sequenced by using primer *ACAA1*-sgRNA-Fc for mutation(s) introduced by the sgRNAs. We obtained two cell clones with an insertion of adenine (c.184-185insA) that disrupted the translation of the ACAA1 protein and the KO of endogenous ACAA1 protein could be validated by western blot.

### Assays for ACAA1 enzymatic activity and lysosomal activity

The enzymatic activities of ACAA1 WT and ACAA1 p.N299S were measured using the Fluorometric Acetyltransferase Activity Assay Kit (Abcam, ab204536), as previously described.^35^ Briefly, the assay was performed with 5 μg of pure protein and 100 nM acetoacetyl coenzyme A sodium salt hydrate (Sigma, A1625), and fluorescence was detected at Ex/Em of 380/520 nm. Both ACAA1 WT and ACAA1 p.N299S proteins were extracted using TnT® Quick Coupled Transcription/Translation Systems (Promega, L1170) and purified using the His-tag Protein Purification Kit (Beyotime, P2226). Each sample was analyzed in triplicate.

Lysosomal activities were determined by using the NAG assay. Briefly, lysates of HM, HM ACAA1 KO, U251-APP, or U251-APP ACAA1 KO cells treated with or without the indicated chemicals or transfected with the indicated expression vectors were isolated. The NAG assay was performed by using a commercial kit from MIBio (Cat. #SU-B16484) following the manufacturer’s instructions.

### Mouse models, AAV-mediated gene delivery, behavioral tests, and tissue analyses

The APP/PS1ΔE9 mice were originally introduced from Jackson Laboratory.^47^ The APP/PS1ΔE9 mice and WT littermates were bred and maintained at the experimental animal core facility of KIZ on a 12-h light/dark cycle, with free access to food and water. In all experiments, genotypes of both *APP* and *PSEN1* were confirmed by using tail DNA following the standard PCR condition.^54^ Animals were divided into sex- and age-matched groups, and both genders were used for analyses.

We used 3-month-old APP/PS1ΔE9 mice and WT littermates for AAV-mediated gene delivery. Briefly, **t**he recombinant AAV php.eb vectors with GFP expression (AAV pAV-C-GFP) carrying empty vector (AAV-Vector), ACAA1 WT (AAV-ACAA1 WT) or p.N299S (AAV-ACAA1 N299S) (Supplementaty Fig. S6a) were developed by the VIGENE BIOSCIENCES.INC. The original titers of AAV-Vector, AAV-ACAA1 WT, and AAV-ACAA1 N299S were 8.52 × 10^13^, 6.68 × 10^13^, and 1.01 × 10^14^ vector genomes (vg)/mL, respectively. The viruses were stored at −80 °C and diluted with saline (0.9% sodium chloride) to 5.00 × 10^13^ vg/mL for injection. Mice were anesthetized by intraperitoneal injection of pentobarbital (0.06 g/kg body weight) and positioned on a stereotactic frame (Panlab HARVARD, MA, USA), then each animal was bilaterally injected with 1 µL viral solution (5.00 × 10^13^ vg/mL) into the hippocampus (stereotaxic coordinates: anteroposterior, −2 mm; mediolateral, ±2.1 mm; dorsoventral, −1.9 mm) with a syringe pump (Panlab, Harvard, MA, USA) at a speed of 200 nL/min. We left the needle in place for an additional 5 min before it was slowly removed. We assessed the effects of AAV-Vector, AAV-ACAA1 WT, and AAV-ACAA1 N299S on behavioral performance in these animals after AAV delivery for 5 months.

The behavioral tests of mice were performed following the previously described protocols.^53–55^ For all behavioral tests, the experimenter was blinded to the genotypes of mice. The detailed information of each test can be found in the online Supplementary Materials and Methods.

After behavioral tests, animals (at an age of 9 months) were euthanized for collecting brain tissues. Briefly, the brain was gently removed and rinsed in cold phosphate-buffered saline (PBS; pH 7.4), followed by immediate dissection into two halves. One half was stored at −80 °C for biochemistry assays, whereas the other half was fixed in 4% paraformaldehyde in PBS at 4 °C for immunohistochemistry and immunofluorescence assays following the previously reported protocol.^54,106^ We followed the previously reported protocols to isolate plaque-related insoluble and soluble Aβ in brain tissues for quantification by ELISA.^54,109^ The detailed information for brain dissection, immunohistochemistry, immunofluorescence, and ELISA for Aβ can be found in the online Supplementary Materials and Methods.

The animal care and experimental protocols were approved by the Institutional Animal Care and Use Committee of KIZ, CAS.

### H&E staining and Nissl staining

The brain tissues were paraffin embedded for cutting into 4-μm sections. Each section was deparaffinized by passing through 100% xylene, then rehydrated through incubation with serial dilutions (100, 95, and 75%) of ethanol (each 10 min). H&E staining and Nissl staining were performed according to the instructions provided by Beyotime Institute of Biotechnology (C0105) and Servicebio (G1032), respectively.

### Electrophysiology in slice cultures

The electrophysiology in brain slice cultures was performed following our previously described protocol.^21,62^ The detailed information can be found in the online Supplementary Materials and Methods.

### Statistical analysis

The number of samples or animals is specified in the caption for each experiment. Specific statistical analyses were performed according to the requirements of different experimental procedures. For comparison of multiple groups, one-way analysis of variance (ANOVA) followed by Tukey’s post hoc tests were used. For average comparison between two groups, we used two-tailed Student’s *t* test. Two-way ANOVA tests were performed to assess the effect on a dependent variable with two independent variables, e.g., time and phenotype. Significant differences in the mean were claimed when *P* < 0.05, with four degrees of significance (*P < 0.05, **P < 0.01, ***P < 0.001, and ****P < 1 × 10^−4^).

## Supplementary information

ACAA1 in AD Supplementary data-Luo-Yao 2021-8-17

## Data Availability

The RNA-sequencing data have been deposited in the GSA database (accession number HRA000978) and are available at the AlzData webserver (http://www.alzdata.org/file/ACAA1.RNAseq.tar.gz).
