# Supplementary material for: A novel missense variant in ACAA1 contributes to early-onset Alzheimer’s disease, impairs lysosomal function, and facilitates amyloid-β pathology and cognitive decline
Source: Signal Transduct Target Ther. 2021 Aug 31;6:325. doi: 10.1038/s41392-021-00748-4 (PMC8408221; doi:10.1038/s41392-021-00748-4)
Supplement: Supplementary file 1 — ACAA1 in AD Supplementary data-Luo-Yao 2021-8-17 [file 41392_2021_748_MOESM1_ESM.docx]

**Supplementary Materials for**

**A novel missense variant in ACAA1 contributes to early-onset Alzheimer’s disease, impairs lysosomal function and facilitates amyloid-β pathology and cognitive decline**

Rongcan Luo ^1,#,*^, Yu Fan ^1,#^, Jing Yang ^1,2^, Maosen Ye ^1,2^, Deng-Feng Zhang ^1^, Kun Guo ^3^, Xiao Li ^1,2^, Rui Bi ^1^, Min Xu ^1^, Lu-Xiu Yang ^1^, Yu Li ^1,2^, Xiaoqian Ran ^1,2^, Hong-Yan Jiang ^4^, Chen Zhang ^5^, Liwen Tan ^6^, Nengyin Sheng ^3,7^, Yong-Gang Yao ^1,2,8,*^

^1^ Key Laboratory of Animal Models and Human Disease Mechanisms of the Chinese Academy of Sciences & Yunnan Province, and KIZ-CUHK Joint Laboratory of Bioresources and Molecular Research in Common Diseases, Kunming Institute of Zoology, Chinese Academy of Sciences, Kunming, Yunnan 650204, China

^2^ Kunming College of Life Science, University of Chinese Academy of Sciences, Kunming, Yunnan 650204, China

^3^ State Key Laboratory of Genetic Resources and Evolution, Kunming Institute of Zoology, Chinese Academy of Sciences, Kunming 650201, China

^4^ Department of Psychiatry, the First Affiliated Hospital of Kunming Medical University, Kunming 650032, China

^5^ Division of Mood Disorders, Shanghai Mental Health Center, Shanghai Jiao Tong University School of Medicine, Shanghai 200030, China

^6^ Mental Health Institute of the Second Xiangya Hospital, Central South University, Changsha 410011, China

^7^ Center for Excellence in Animal Evolution and Genetics, Chinese Academy of Sciences, Kunming, Yunnan 650201, China

^8^ CAS Center for Excellence in Brain Science and Intelligence Technology, Chinese Academy of Sciences, Shanghai 200031, China

^#^ These authors contributed equally to this work

^∗^ Corresponding author, E-mail addresses: yaoyg@mail.kiz.ac.cn (Y.-G. Yao), luorongcan@mail.kiz.ac.cn (R. Luo)

**This PDF file includes:**

Supplementary Materials and Methods

Figures S1 to S7

Tables S1 to S4

Supplementary Materials and Methods

**Cell culture and transfection**

The U251 glioma cells, human microglia (HM), SH-SY5Y neuroblastoma cells were introduced from Kunming Cell Bank, Kunming Institute of Zoology (KIZ), Chinese Academy of Sciences (CAS). We introduced human APP mutant K670N/M671L into U251 cells for stable expression (U251-APP cell), and this cell line produced Aβ under doxorubicin induction ^1,2^. We overexpressed ACAA1 WT and ACAA1 p.N299S in the U251-APP cells and HM cells to characterize their potential effects. Briefly, the U251-APP cells and HM cells were cultured in Roswell RPMI-1640 medium and Dulbecco’s modified Eagle’s medium (DMEM; Gibco-BRL, 11965-092), respectively, supplemented with 10% fetal bovine serum (FBS) (Gibco-BRL; 10099-141), 100 U/ml penicillin and 100 mg/mL streptomycin at 37^o^C in a humidified atmosphere incubator with 5% CO_2_. Transfection of empty vector, expression vector of ACAA1 WT, and expression vector of ACAA1 p.N299S in U251-APP cells or HM cells, was performed using an electroporator (CUY21EDIT, Nepa gene Co., Japan) following the manufacturer’s instructions. After cells were trypsinized and washed three times with Opti-MEM medium (Gibco-BRL), around 1 × 10^6^ cells were resuspended in 100 μL Opti-MEM medium, and were electroporated with 10 μg plasmids. Transfected cells were seeded in pre-warmed growth medium for 72 h in 5% CO2 at 37^o^C before the harvest.

The SH-SY5Y cells were maintained in DMEM supplemented with 10% FBS, 1×MEM nonessential amino acid solution (Gibco, 11140050), 100 U/ml penicillin and 100 mg/ml streptomycin at 37^o^C in a humidified atmosphere incubator with 5% CO_2_. For knockdown of the *ACAA1* gene in this cell line, three small interfering RNAs (siRNAs: siRNA-1, 5’-GAGCAAGGGCTGTTTCCAA-3’; siRNA-2, 5’-CCGCTGCCGTCTTTGAATA-3’; siRNA-3, 5’-TGAGCGGTTTGGCATTTCA-3’) targeting *ACAA1* and a negative control siRNA (NC siRNA) were synthesized by RiboBio (Guangzhou, China). The SH-SY5Y cells (2 × 10^5^ per well) were seeded in 6-well plates to grow to 50% confluence, then culture medium was removed and washed once with the Opti-MEM medium before the transfection. The *ACAA1* siRNA or NC siRNA was dissolved in the Opti-MEM medium, then was mixed with 6 μL Lipofectamine^TM^ 3000 (Invitrogen, L3000008) to achieve a final volume of 150 μL for forming the siRNA-Lipofectamine mixture for 20 min at room temperature. The mixture was added to each well, together with an additional 850 μL Opti-MEM medium. After an incubation of 6 h, the medium was removed and 2 mL fresh medium was added to each well for growth. We used three siRNA concentrations (12.5 nM, 25 nM, 50 nM) to optimize the knockdown efficiency, and transfected cells were harvested at 48 h. Transfection of empty vector and expression vector of ACAA1 WT or mutant p.N299S was performed by using the Lipofectamine^TM^ 3000 with a similar procedure as for siRNA transfection.

**Evolutionary conservation analysis**

Evolutionary conservation analysis was performed to show the overall sequence identity of the ACAA1 protein sequences. We retrieved the ACAA1 protein sequences of 13 vertebrate species, including human (*Homo sapiens*, ID: 30), chimpanzee (*Pan troglodytes*, ID: 460268 ), gorilla (*Gorilla gorilla gorilla*, ID: 101141231), pig-tailed macaque (*Macaca nemestrina*, ID: 105480193), Chinese tree shrew (*Tupaia chinensis*, ID: 102470441), house mouse (*Mus musculus*, ID: 113868), norway rat (*Rattus norvegicus*, ID: 24157), pig (*Sus scrofa*, ID: 100515577), cattle (*Bos taurus*, ID: 508324), dog (*Canis lupus familiaris*, ID: 477023), domestic cat (*felis catus*, ID: 101087343), chicken (*Gallus gallus*, ID:770094), and zebrafish (*danio rerio*, ID: 431754), from NCBI ([http://www.ncbi.nlm.nih.gov](http://www.ncbi.nlm.nih.gov/pubmed)). Protein sequence alignment was performed by ClusterW method using software MEGA 7.0 ^3^.

**RNA-sequencing (RNA-seq) analysis**

We followed the procedure in our previous study for the RNA-seq analysis ^4^. In brief, we extracted total RNA from transfected U251-APP cells and HM cells by using the RNA isolation kit (TianGen, Co. Ltd.). We only used RNA samples with a high quality, as reflected by an A260/A280 ratio of 1.8-2.0 to construct library for RNA-seq. Sequencing libraries were generated using the NEBNextUltraTM RNA Library Prep kit for Illumina (NEB, USA) following the manufacturer’s recommendation, and were sequenced on an Illumina-HiSeq 4000 platform. The raw sequencing reads (150 bp paired-ends reads) were first trimmed by Trimmomatic (version 0.38) software ^5^ to remove the adapter sequences and low-quality sequences using the following parameters “LEADING:3 TRAILING:3 SLIDINGWINDOW:4:15 MINLEN:36”. After reads trimming, the clean reads were aligned to the human reference genome GRCh38.p13 (https://www.ncbi.nlm.nih.gov/assembly/GCF_000001405.39) using STAR software (version 2.6.0c) ^6^. Next, the aligned reads in bam format generated by STAR were subjected to featureCounts^7^ to assign and count the uniquely mapped fragments to exons according to the annotation file of GRCh38.p13. We used rlogTransformation function of R package DESeq2 ^8^ to normalize and scale reads counts generated in the previous step. Principal component analysis and differential expression analysis were performed using the DESeq2 ^8^ based on the normalized count. The *P* values were adjusted (*P*_adjust_) by the Benjamini & Hochberg (BH) method. We defined differentially expressed genes (DEGs) between different conditions if *P*_adjust_ < 0.05. Gene Ontology (GO) ^9^ and Kyoto Encyclopedia of Genes and Genomes (KEGG) ^10^ pathway enrichment analyses were performed for DEGs using R package clusterProfiler ^11^. The gene set enrichment analysis (GSEA) ^12^ was performed based on the log_2_ fold change of DEGs obtained from differential gene analysis, and all molecular signature databases provided by MSigDB (https://www.gsea-msigdb.org/gsea/msigdb/index.jsp) were tested for enrichment.

**Western blotting**

Western blotting was performed as described in our previous studies ^13,14^. In brief, lysates of different mouse brain tissues, transfected HM cells, U251-APP cells and SH-SY5Y cells were prepared using the protein lysis buffer (Beyotime Institute of Biotechnology, P0013) with phosphatase and protease inhibitors (Abcam, ab201119). We measured protein concentration of each sample by using the BCA protein assay kit (Beyotime Institute of Biotechnology, P0012). Around 20 μg protein was separated by 12% SDS PAGE, and was transferred to a polyvinylidene difluoride membrane (Bio-Rad, L1620177 Rev D). After the membrane was soaked with 5% (w/v) skim milk for 2 h at room temperature, membrane was incubated with the respective primary antibodies overnight at 4°C. The membrane was washed 3 times with Tris-buffered saline with 0.1% Tween 20 (TBST), each time 5 min, followed by incubation with the peroxidase-conjugated anti-mouse or anti-rabbit IgG (1:10000; KPL) for 1 h at room temperature. The membrane was visualized using an ECL Western blot detection kit (Millipore, WBKLS0500). ImageJ (National Institutes of Health, Bethesda, Maryland, USA) was used to evaluate the densitometry. Western blot for ACTB, β-tubulin, or GAPDH was used as a loading control.

**Electrophysiology in slice cultures**

The electrophysiology in brain slice cultures was performed following the previously described protocol ^15,16^. Briefly, organotypic rat hippocampal slice cultures were made from postnatal day 6-8 (P6-P8) rat. The ACAA1 WT and ACAA1 p.N299S were subcloned into the pCAGGS vector harboring EGFP. Biolistic transfections were performed on day in vitro (DIV) 2 after culture using a Helios Gene Gun (Bio-Rad) with 1 μm DNA-coated gold particles. Slices were maintained at 34 °C with medium changes every other day. On DIV 8, voltage-clamp dual whole-cell recordings for CA1 pyramidal neurons were taken from a fluorescent transfected neuron and a neighboring un-transfected control neuron. During recording, slices were transferred to a perfusion stage on an Olympus BX51WI upright microscope and perfused at 2.5 mL/min with artificial cerebrospinal fluid (ACSF: 119 mM NaCl, 2.5 mM KCl, 4 mM CaCl_2_, 4 mM MgSO_4_, 1 mM NaH_2_PO_4_, 26.2 mM NaHCO_3_, and 11 mM glucose) bubbled with 95% O_2_ and 5% CO_2_. Series resistance was monitored on-line. We discarded recordings in these series increased to >30 MOhm or varied by >50% between neurons. Dual whole-cell recordings measuring evoked excitatory postsynaptic currents (EPSCs) were performed. When measuring EPSCs, 100 μM picrotoxin was added to block inhibitory currents and 4 μM 2-Chloroadenosine was used to control epileptiform activity. Internal solution was composed of 135 mM CsMeSO_4_, 8 mM NaCl, 10 mM HEPES, 0.3 mM EGTA, 5 mM QX314-Cl, 4 mM MgATP, 0.3 mM Na_3_GTP, and 0.1 mM spermine. A bipolar stimulation electrode was placed in stratum radiatum, and responses were evoked at 0.2 Hz. Peak AMPAR responses were recorded at -70 mV, and NMDAR responses were recorded at +40 mV, with amplitudes measured 100 ms after stimulation to avoid contamination by AMPAR current. Paired-pulse ratio was determined by delivering two stimuli 40 ms apart and dividing the peak response to stimulus 2 by the peak response to stimulus 1. Peak GABA currents were recorded at 0 mV. All the data were analyzed off-line with custom software (IGOR Pro). Responses were collected with a Multiclamp 700A amplifier (Axon Instruments), filtered at 2 kHz, and digitized at 10 kHz.

**Behavioral tests**

The locomotor activity of mice in the open-field test were recorded and tracked using the SMART 3.0 software (Panlab HARVARD, MA, USA), as previously described ^17^ with slight modifications. In brief, mice were individually placed in the center of an open-top chamber (40 × 40 × 40 inches) with an array of photobeams around the periphery, and allowed to explore the chamber for 15 min each day for 3 consecutive days. Locomotor activity in the three training trials was recorded by the photobeams, and the distance moved was recorded. We cleaned the chamber with 70% ethanol after each trial.

The contextual fear conditioning behavioral assessment was performed as previously described ^17^. On the 1^st^ day of training, mice were allowed to explore in an enclosed training chamber with the floor wired to an electric shock generator for 180 s. The animals were then exposed to a pure tone for 30 s, followed by a 2-s foot shock (0.8 mA). At 60 s after delivery of the shock, mice were returned to their home cages. The fear response (freezing) was assessed at the 1^st^ day and the 7^th^ day after the electric shock. Mice were re-exposed in the original chamber for 8 min (context test), and the time of freezing was measured using the Ugo Basile Fear Conditioning System (UGO).

Morris water maze behavioral assessment was performed as previously described ^13,18^. Briefly, experiments were performed in a 120-cm diameter, 50-cm deep tank filled with opacified water at 20 ± 1°C. The tank was equipped with a 10-cm diameter platform submerged 1 cm under the water surface. Training course contained daily sessions (3 trials per session) for 7 consecutive days. The start positions varied pseudorandomly among the 4 cardinal points. Mean intertrial interval was 90 min. We ended each trial when the animal reached the platform. A 60 sec cut-off was used, after which mice were gently guided to the platform. Once on the platform, animals were given a rest for 20 s before being returned to their cage. Four h (short-term memory) and 72 h after the last training trial (day 10), retention was assessed during probe trial in which the platform was removed. Animals were video-tracked using SMART 3.0 software (Panlab HARVARD, MA, USA). Behavioral parameters, including swim speed, travelled distance, latency, percentage of time in each quadrant, percentage of distance in each quadrant, were automatically calculated. For all behavioral tests, the experimenter was blinded to the genotypes of mice.

**Brain dissection, immunohistochemistry, and immunofluorescence**

The APP/PS1ΔE9 mice and WT littermates with AAV-mediated gene delivery were sacrificed at 6 months post-injection. Following anesthesia, mice were transcardially perfused with ice-cold 0.1 M PBS (pH 7.4) before dissection. The left cerebral hemisphere was dissected and post-fixed in 4 % PFA for 1 week, followed by embedded in paraffin wax for immunohistochemistry analysis or in optimal cutting temperature (OCT) compound for immunohistochemistry and immunofluorescence assays. The paraffin-embedded brain slices (4 μm thickness) were cut using a microtome (Leica, Germany) and stored at room temperature. The OCT-embedded brain slices (at 10 μm thickness) were cut using a freezing microtome (Leica, Germany) and stored at -80 °C. Each brain slice of APP/PS1ΔE9 mice was incubated with mouse anti-β-amyloid (antibody 4G8; 1:500; BioLegend, 800701) overnight at 4°C. After 3 washes with PBS, the brain slice was incubated with horseradish peroxidase-labeled goat anti-mouse (1:200; Servicebio, GB23301) for 1 h at room temperature. Images were acquired by scanning with the Pannoramic Digital Slide Scanner (3DHISTECH) and were processed with CaseViewer software (3DHISTECH). The staining intensity was quantified by using ImageJ software (National Institutes of Health, Bethesda, Maryland, USA).

We followed the previously reported protocol for immunofluorescence assays ^13,14^. In brief, after antigen retrieval, brain sections were blocked in PBS supplemented with 5% BSA for 1 h. Sections were then incubated with the respective primary antibodies (**Table S4**) overnight at 4°C. After washing with PBS, sections were incubated with the respective secondary antibodies (**Table S4**). The sections were then subjected to DAPI staining (1:1000; Invitrogen, D1306) for 15 min, before being sealed with antifade mounting medium (Beyotime Institute of Biotechnology, P0128M) and glass covers. Images were acquired using the same procedure and scanner as above. Counts of NeuN-positive cells were assessed for the degree of neuronal loss in the hippocampal CA3 region. Quantification of the number and area of Aβ plaques was performed using the particle analysis tool of the ImageJ software as previously described ^19^.

**ELISA for Aβ**

We followed the previously reported protocols to isolate plaque-related insoluble and soluble Aβ ^13,20^. In brief, hippocampus and cortex tissues from APP/PS1ΔE9 mice were stored at -80°C after collection. Upon use, each sample was homogenized with 300 μL of RIPA lysis buffer (Beyotime Institute of Biotechnology, P0013) containing protease and phosphatase inhibitors (Abcam, ab201119) on ice, followed by centrifugation at 13,000 g for 15 min at 4°C to collect supernatants and pellets, respectively. The protein concentrations of the supernatants were measured by using BCA protein assay kit and were adjusted to same concentration for quantifying soluble Aβ by ELISA. The pellets were resuspended in a volume of 300 μL of RIPA lysis buffer, followed by centrifugation at 13,000g for 15 min at 4°C to remove potential soluble Aβ. This washing step was repeated 3 times to remove all soluble Aβ. Then, the pellets containing insoluble Aβ were solubilized in sodium dodecyl sulfate (SDS) buffer (2% SDS, 25 mM Tris-HCl, pH 7.4) ^13,20^. After pulsed sonication for 15 sec, the SDS fractions were quantified for protein concentrations and processed for measuring insoluble Aβ, using the same procedure for soluble Aβ. The levels of Aβ40/Aβ1-40 and Aβ42/Aβ1-42 in mouse brain tissues and U251-APP cell culture supernatant were determined by using Aβ40 kit (Elabscience, E-EL-H0542c) and Aβ42 kit (Elabscience, E-EL-H0543c), respectively.


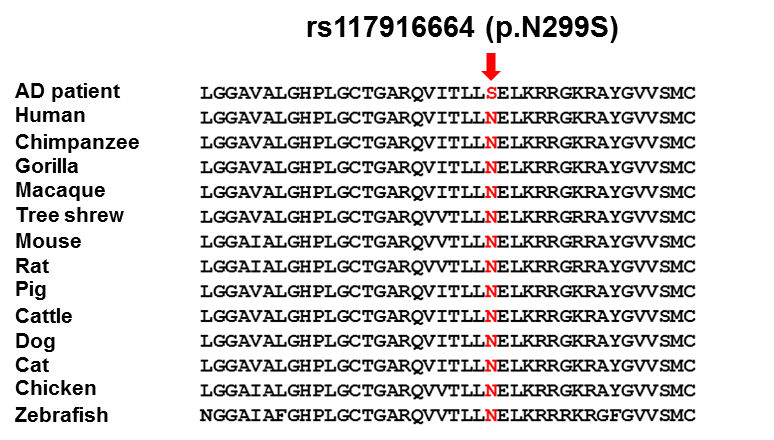


**Figure S1. Evolutionary conservation of ACAA1 p.N299S.** Alignment of ACAA1 protein sequences from different species. The ACAA1 protein sequences of human (*Homo sapiens*, ID: 30), chimpanzee (*Pan troglodytes*, ID: 460268 ), gorilla (*Gorilla gorilla gorilla*, ID: 101141231), pig-tailed macaque (*Macaca nemestrina*, ID: 105480193), Chinese tree shrew (*Tupaia chinensis*, ID: 102470441), house mouse (*Mus musculus*, ID: 113868), rat (*Rattus norvegicus*, ID: 24157), pig (*Sus scrofa*, ID: 100515577), cattle (*Bos taurus*, ID: 508324), dog (*Canis lupus familiaris*, ID: 477023), cat (*felis catus*, ID: 101087343), chicken (*Gallus gallus*, ID:770094), and zebrafish (*danio rerio*, ID: 431754), were from NCBI ([http://www.ncbi.nlm.nih.gov](http://www.ncbi.nlm.nih.gov/pubmed)).

**
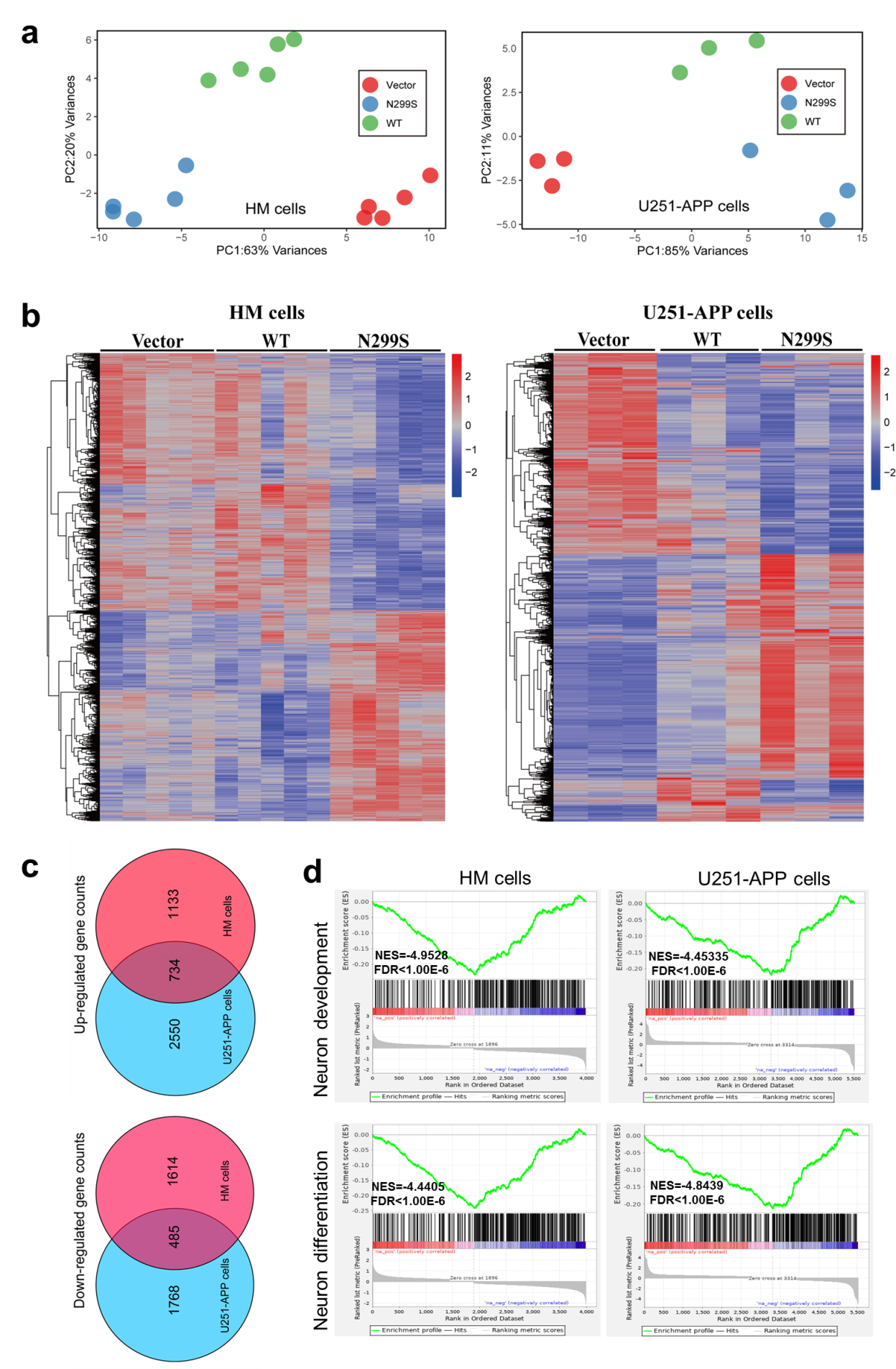
**

**Figure S2. RNA-seq analyses of U251-APP and HM cells with overexpression of ACAA1 WT and ACAA1 p.N299S. (a**) Principal component analysis (PCA) of U251-APP and HM cells with overexpression of wild type ACAA1 (WT) and ACAA1 p.N299S (N299S), as well as the empty vector (Vector). PCA was performed based on the expression values of all expressed genes, and each point represented a sample. (**b**) Expression heatmaps of HM cells (*Left*) and U251-APP cells (*right*) with overexpression of wild type ACAA1 (WT), ACAA1 p.N299S (N299S), and the empty vector (Vector). (**c)** Venn diagram of differentially expressed genes (DEGs) identified in U251-APP and HM cells with overexpression of ACAA1 WT and ACAA1 N299S. (**d**) Gene set enrichment analyses (GSEA) showed the enrichment of neuron development and neuron differentiation in in HM (*left panel*) and U251-APP (*right panel*) cells. NES, normalized enrichment score; FDR, false discovery rate.

**
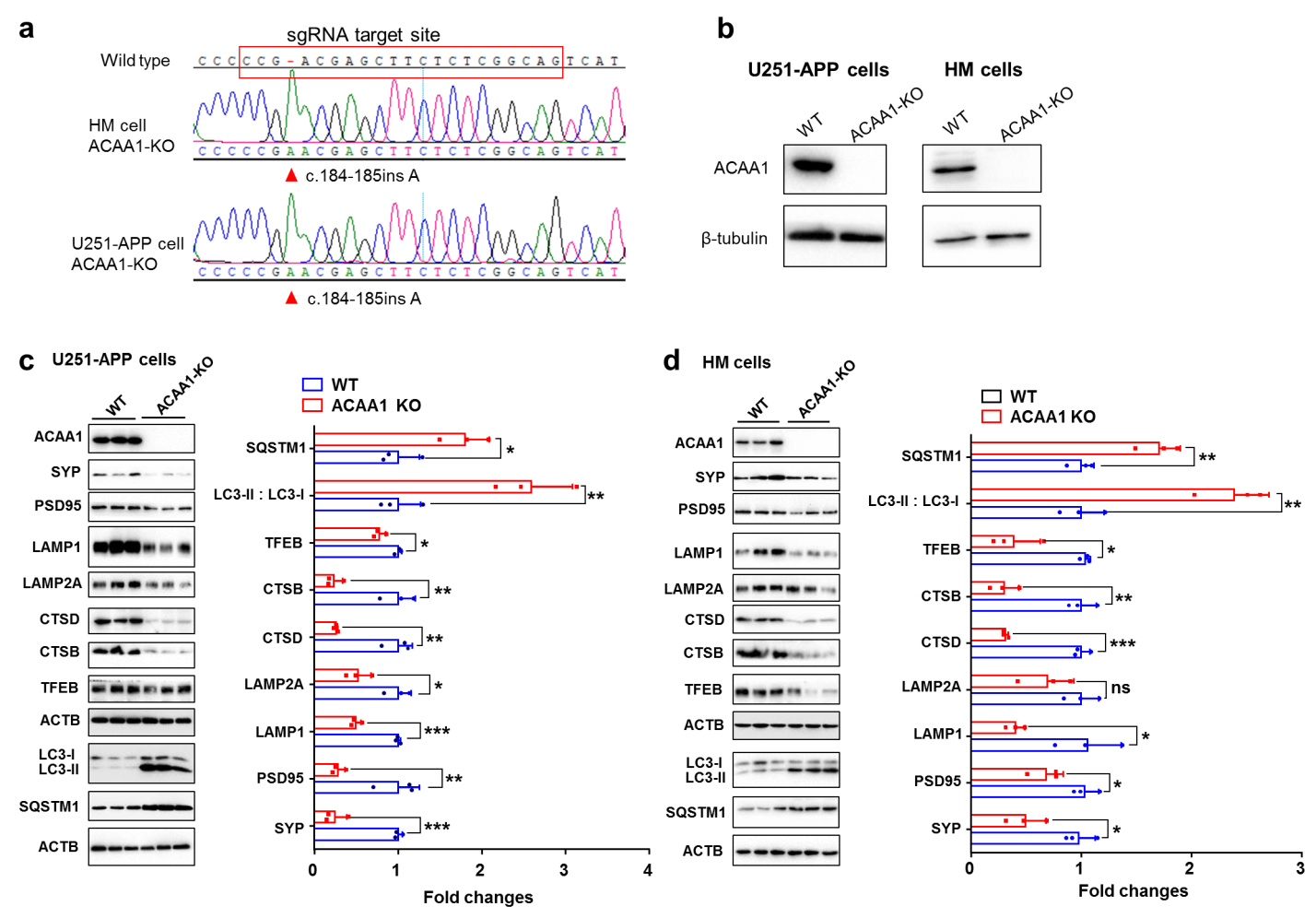
**

**Figure S3. Knockout of ACAA1 in U251-APP and HM cells impaired levels of lysosomal and postsynaptic proteins and affected autophagy.** Successful knockout of the *ACAA1* gene in HM cells and U251-APP cells by using the CRISPR/Cas9 technology (**a-b**). (**a**) Sequencing chromatographs showing the introduced mutations in the *ACAA1* gene in U251-APP and HM cells with successful gene editing (ACAA1-KO) and the unedited cells (WT). (**b**) Absence of the ACAA1 protein in these ACAA1-KO cells. The β-tubulin was used as the loading control. (**c-d**) ACAA1 knockout caused decreased levels of LAMP1, LAMP2A, CTSD, CTSB, TFEB, SYP and PSD95, but increased LC3-II:LC3-I ratio and SQSTM1 protein level in U251-APP (**c**) and HM (**d**) cells. The ACTB was used as the loading control. Data are representative of 3 independent experiments with similar results. ns, not significant; *, *P* < 0.05; **, *P* < 0.01; ***, *P* < 0.001; Students *t* test. Bars represent mean ± SD of the three experiments.


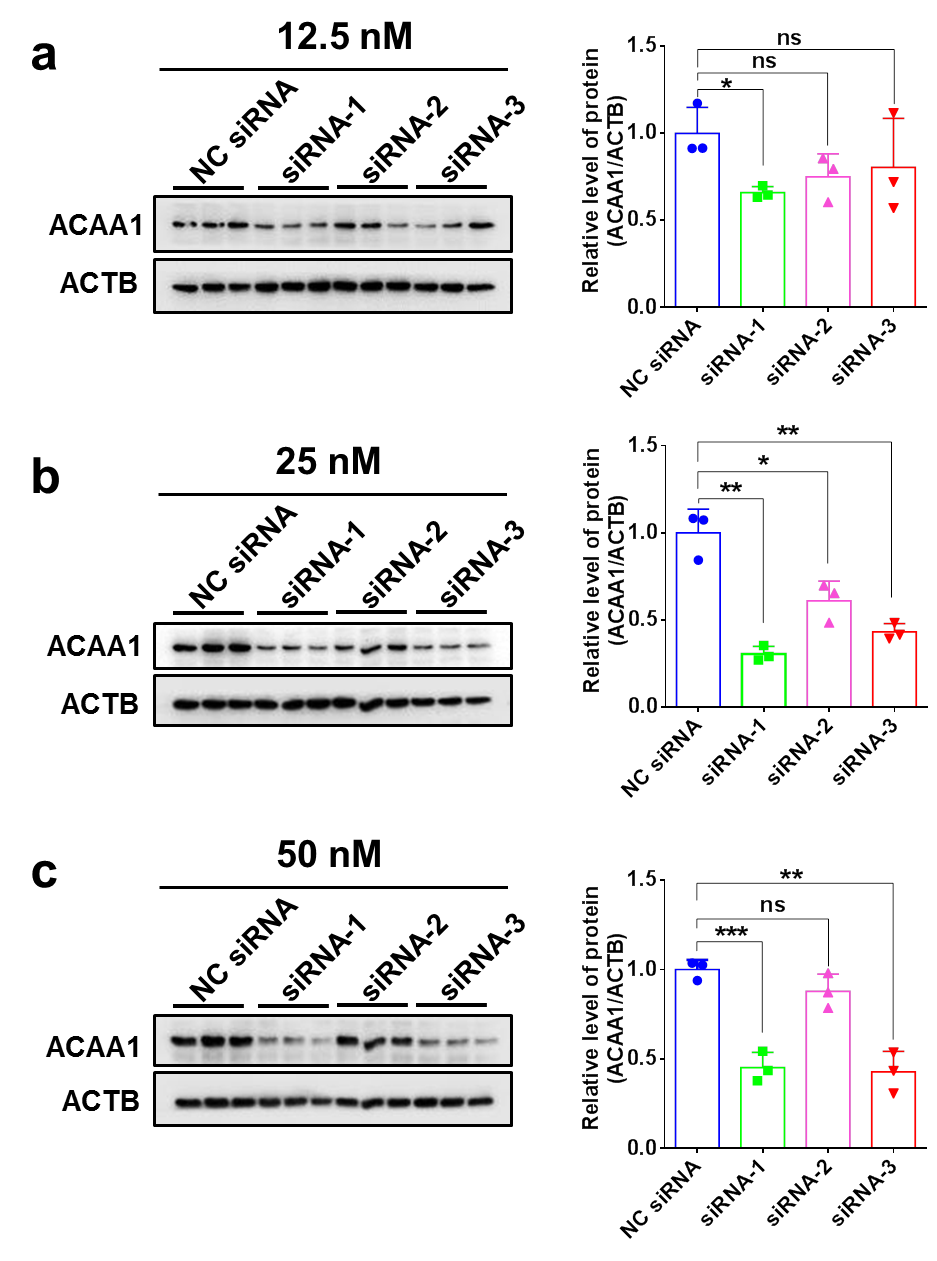


**Figure S4. Knockdown efficiency of the *ACAA1* siRNAs in human SH-SY5Y cells.** Cells were transfected with each of 3 *ACAA1* siRNAs (siRNA-1, siRNA-2 and siRNA-3) and negative control siRNA (NC siRNA), respectively, for 48 h before harvest for Western blotting analysis. The siRNAs were used at different concentrations of 12.5 nM (a), 25 nM (b) and 50 nM (c). Each transfection contains 3 wells in a 6-well plate. Relative protein abundance was normalized to the ACTB. ns, not significant; *, *P*<0.05; **, *P*<0.01; Student’s t test. Bars represent mean ± SD.


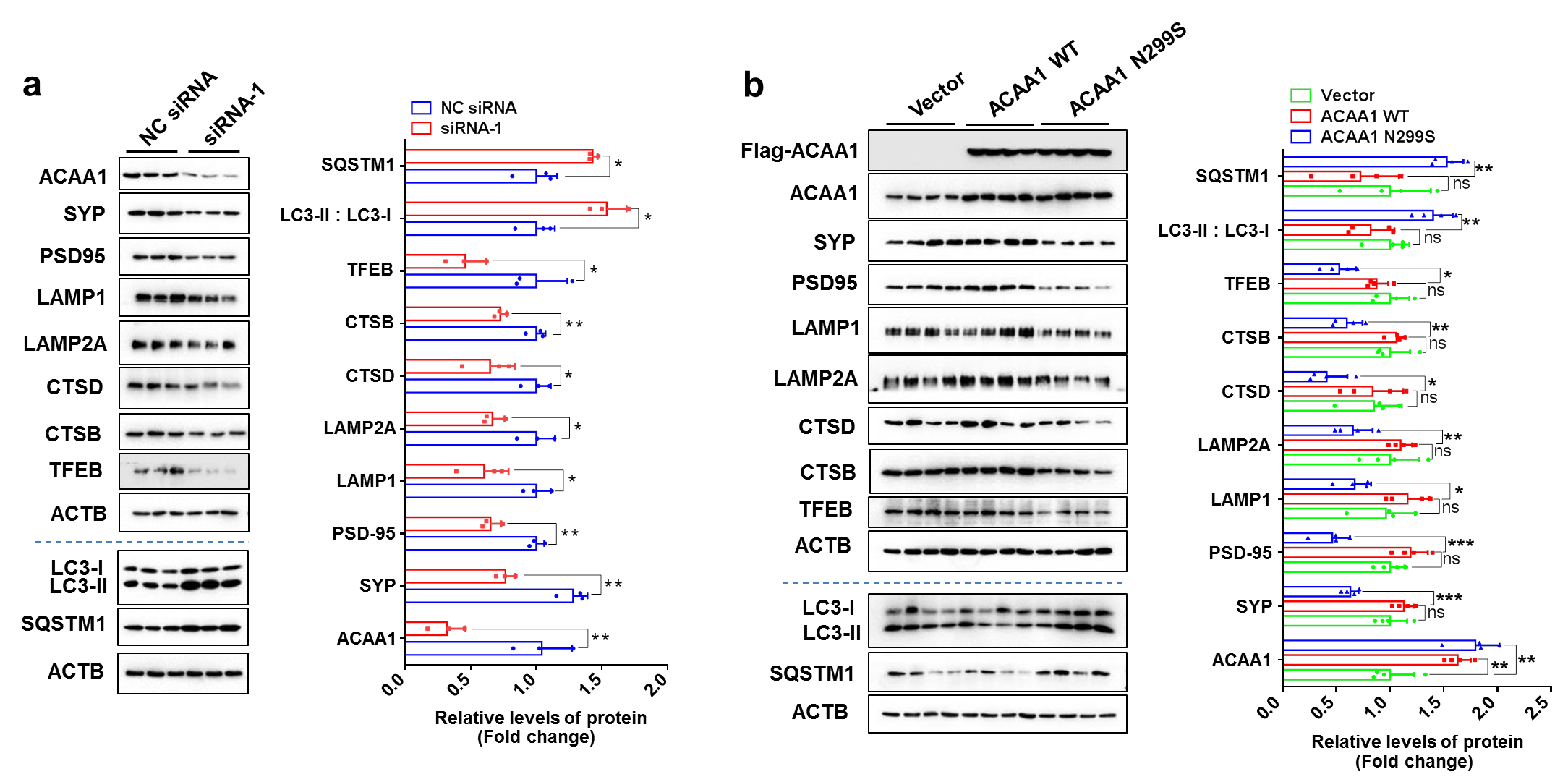


**Figure S5. Knockdown of the *ACAA1* gene or overexpression of ACAA1 p.N299S disturbed the lysosomal and synaptic functions and declined autophagy in SH-SY5Y cells.**

(**a-b**) ACAA1 knockdown (**a**) and overexpression of ACAA1 p.N299S (b) caused decreased lysosomal protein levels of LAMP1, LAMP2A, CTSD, CTSB, TFEB, SYP and PSD95, but increased LC3-II:LC3-1 ratio and SQSTM1 level in SH-SY5Y cells. The ACTB was used as the loading control. Data are representative of 3 independent experiments with similar results. ns, not significant; *, *P* < 0.05; **, *P* < 0.01; ***, *P* < 0.001; Students *t* test. Bars represent mean ± SD of the three experiments.


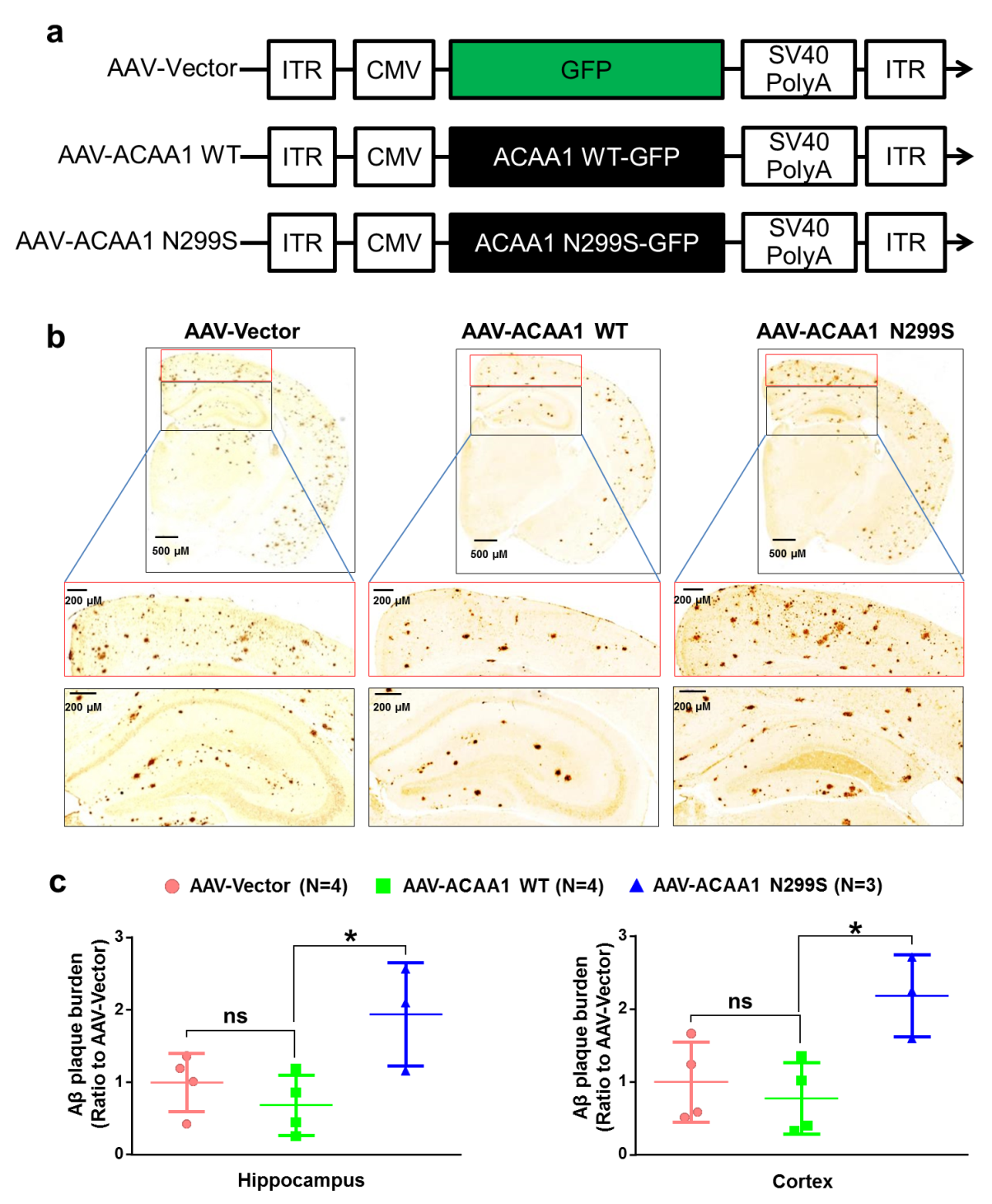


**Figure S6. Administration of ACAA1 p.N299S increased amyloid plaque burden in APP/PS1ΔE9 mice. (a)** A schematic representation of the AAV vector system. The AAV php.eb-CMV-GFP constructs enabling the broad-spectrum expression of GFP (AAV-Vector), GFP-tagged ACAA1 WT (AAV-ACAA1 WT) and ACAA1 p.N299S (AAV-ACAA1 N299S) were constructed. ITR: inverted terminal repeat; GFP: GFP-tag; SV40 PolyA: SV40 poly A signal addition functions to terminate transcription from upstream promoter activity. (**b-c**) Overexpression of ACAA1 p.N299S aggravated amyloid plaque pathology in APP/PS1ΔE9 mice. Representative immunochemistry images (**b**) and quantification of 4G8-stained amyloid plaques in the hippocampus and cortex tissues (**c**) of APP/PS1ΔE9 mice after delivery of AAV-Vector, AAV-ACAA1 WT and AAV-ACAA1 N299S. N = 4 or 3 mice per group. ns, not significant; **P* < 0.05, Student’s *t* test. Bars represent mean ± SD.


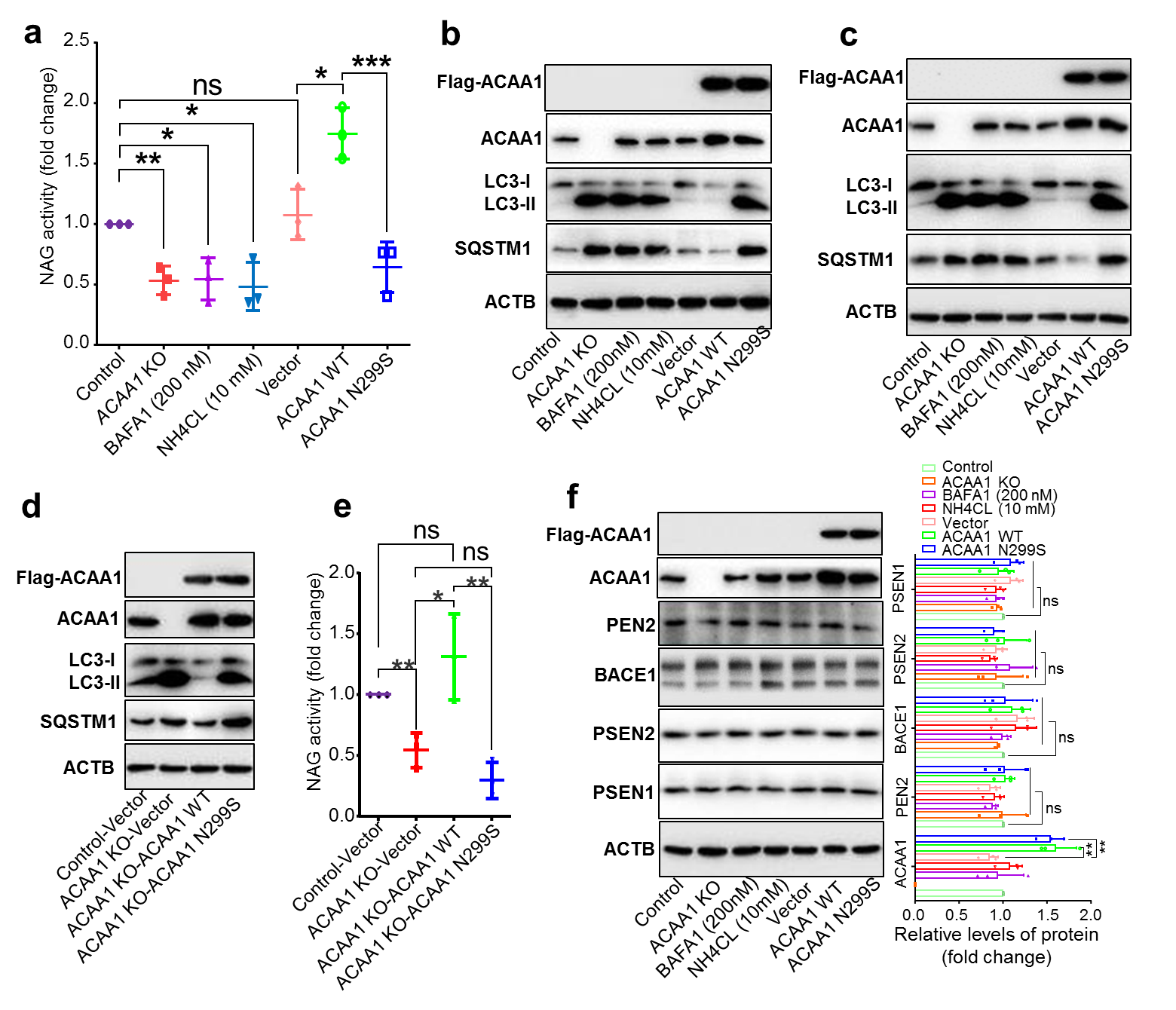


**Figure S7. ACAA1 p.N299S impaired autophagy and lysosomal function in U251-APP and HM cells.** (**a**) Knockout of ACAA1 or overexpression of ACAA1 p.N299S in HM cells led to a reduction of NAG activity, similar to the inhibition effect of BAFA1 and NH4CL treatments. (**b-c**) Knockout of ACAA1 or overexpression of ACAA1 p.N299S affected autophagy in U251-APP cells (**b**) and HM cells (**c**), similar to that of BAFA1 and NH4CL treatments. (d-e) Overexpression of ACAA1 WT, but not ACAA1 p.N299S, rescued the altered autophagy (**d**) and lysosomal activity (**e**) in HM ACAA1 KO cells. (**f**) Knockout of ACAA1 or overexpression of ACAA1 p.N299S had no effect on the protein levels of BACE1, PSEN1, PSEN2 and PEN2 in U251-APP cells. Control, cells without ACAA1 knockout. For chemical treatments, cells treated with BAFA1 at a concentration of 200 nM or NH4CL at a concentration of 10 mM. For transfection, cells were transfected with empty vector (Vector) or expression vectors for ACAA1 WT and ACAA1 p.N299S, respectively. Shown data were representative of three independent experiments. ns, not significant; *, *P* < 0.05; **, *P* < 0.01; ***, *P* < 0.001; Student’s *t* test. Bars represent mean ± SD.

Table S1.

**Rare damaging missense variants in known dementia-causal genes in four individuals of the EOFAD family**

| **Causal genes** | **Gene** | **Chr** | **Position** | **SNP_ID** | **Frequency (1000 Genomes)** | **Ref** | **Function** | **Individuals** | | | | **Damaging Prediction** |
| --- | --- | --- | --- | --- | --- | --- | --- | --- | --- | --- | --- | --- |
|  |  |  |  |  |  |  |  | **EADQ01 (II:1)** | **EADM02 (III:4)** | **EADS03 (II:3, AD)** | **EADX04**  **(III:3)** |  |
| Alzheimer disease | *APP* | - | - | - | - | - | - | - | - | - | - | - |
|  | *PSEN1* | - | - | - | - | - | - | - | - | - | - | - |
|  | *PSEN2* | chr1 | 227075798 | . | NA | C | p.H169N | C/C | C/A | C/C | C/C | 0 |
|  | *UNC5C* | chr4 | 96106322 | rs2289043 | 0.495 | A | p.M721T | G/G | G/G | A/G | G/G | 2 |
| Dementia with Lewy bodies | *LRRK2* | chr12 | 40713901 | rs11564148 | 0.286 | T | p.S1647T | T/A | T/A | T/A | T/T | 0 |
|  | *LRRK2* | chr12 | 40619082 | rs2256408 | 0.969 | G | p.R50H | A/A | A/A | A/A | A/A | 0 |
|  | *LRRK2* | chr12 | 40677699 | rs34410987 | 0.002 | C | p.P755L | C/C | C/C | C/C | C/T | 0 |
|  | *LRRK2* | chr12 | 40657700 | rs7308720 | 0.099 | C | p.N551K | C/G | C/C | C/C | C/C | 3 |
|  | *LRRK2* | chr12 | 40758652 | rs3761863 | 0.552 | T | p.M2397T | C/C | T/C | T/C | T/T | 0 |
|  | *PINK1* | chr1 | 20977000 | rs1043424 | 0.301 | A | p.N521T | A/A | A/A | A/C | A/C | 0 |
|  | *PINK1* | chr1 | 20972111 | rs3738136 | 0.123 | G | p.A340T | G/G | G/G | G/G | G/A | 0 |
| Vascular dementia | *COL4A1* | chr13 | 110839550 | rs536174 | 1.000 | T | p.T555P | G/G | G/G | G/G | G/G | 0 |
|  | *COL4A1* | chr13 | 110959356 | rs9515185 | 0.424 | C | p.V7L | C/G | G/G | C/G | C/G | 0 |
|  | *COL4A1* | chr13 | 110818598 | rs3742207 | 0.288 | T | p.Q1334H | T/T | T/T | T/G | T/G | 0 |
|  | *NOTCH3* | chr19 | 15271771 | rs1044009 | 0.629 | G | p.A2223V | A/A | A/A | A/A | G/A | 0 |
| Other neurodegenerative disorders | *DNMT1* | chr19 | 10291113 | rs75616428 | 0.008 | C | p.V120L | C/C | C/C | C/C | C/G | 0 |
|  | *DNMT1* | chr19 | 10273372 | rs2228612 | 0.198 | T | p.I311V | T/C | T/C | T/C | C/C | 0 |
|  | *DNMT1* | chr19 | 10291181 | rs16999593 | 0.045 | T | p.H97R | T/C | T/C | T/C | T/C | 0 |
|  | *CSF1R* | chr5 | 149450132 | rs10079250 | 0.153 | T | p.H362R | T/C | T/C | C/C | T/C | 1 |

Causal genes were defined as described in our previous study ^21^. We detected all the rare (MAF≤0.01 in the dataset if the 1000 Genomes Project ^22,23^), inherited loss-of-function (i.e. stop-gain or frameshift) and damaging missense variants in the four individuals underwent whole genome sequencing. Damaging prediction was scored by the number of prediction algorithms (SIFT ^24^, PolyPhen2 HumDiv, PolyPhen2 HumVar ^25,26^, LRT ^27^ and MutationTaster ^28^) by which the variant was predicted to be damaging. Missense variants were rated as damaging when at least two of the five predictions suggested a potential deleterious effect. “0” means none of the five prediction algorithms suggesting a damaging effect. There are no rare or damaging variants in the *APP* and *PSEN1* genes in the four individuals, and was marked with “-“.

Chr, chromosome; Position, physical position of the variant; SNP_ID, rsID in dbSNP (https://www.ncbi.nlm.nih.gov/snp/); Ref, reference allele, allele of reference genome hg19 (also known as GRCh37, https://grch37.ensembl.org/Homo_sapiens).

Table S2.

**Validation of the rare damaging variants in the EOFAD family using whole-exome data of Chinese patients with EOFAD**

| Chr | Position | SNP_ID | Gene | Residue Change | MAF_1000 Genomes | Reference allele | EADM02 (III:4) | EADQ01 (II:1) | EADS03 (II:3, AD) | EADX04 (III:3) | AD_AC | AD_AN | ctrl_AC | ctrl_  AN | *Fisher P* | OR |
| --- | --- | --- | --- | --- | --- | --- | --- | --- | --- | --- | --- | --- | --- | --- | --- | --- |
| chr19 | 45411941 | rs429358 | APOE | C130R | 0.1506 | C | T/C | T/C | T/T | T/C | 80 | 332 | 61 | 736 | 1.12E-11 | 3.51 |
| chr3 | 38167080 | rs117916664 | ACAA1 | N299S | 0.0038 | T | T/C | T/C | C/C | T/C | 17 | 324 | 15 | 736 | **9.85E-3** | 2.66 |
| chr4 | 106158550 | rs141975400 | TET2 | E1151* | 0.0012 | G | G/G | G/G | G/T | G/G | 2 | 212 | 3 | 576 | 0.62 | 1.82 |
| chr17 | 33550428 | rs537835860 | TBC1D3D | K25* | NA | T | T/T | T/T | T/A | T/T | 1 | 182 | 7 | 736 | 1.00 | 0.58 |
| chr11 | 48266736 | rs7120775 | OR4X2 | Y27* | 0.0028 | C | C/C | C/C | C/G | C/C | 57 | 320 | NA | NA | 1.00 | NA |
| chrX | 14027177 | rs3747421 | GEMIN8 | E195V | 0.0050 | T | T/A | T/A | A/A | T/T | 45 | 318 | NA | NA | 1.00 | NA |
| chrX | 32404572 | rs72468638 | DMD | K1510R | 0.0014 | T | T/C | T/C | C/C | T/T | 4 | 336 | NA | NA | 1.00 | NA |
| chr5 | 1240757 | rs7447815 | SLC6A18 | Y319* | 0.0006 | C | C/G | G/G | G/G | C/G | 110 | 332 | NA | NA | 1.00 | NA |
| chrX | 135426968 | rs5930931 | GPR112 | P368H | 0.0001 | C | C/A | C/A | A/A | C/C | 81 | 318 | NA | NA | 1.00 | NA |
| chr19 | 43698682 | - | PSG4 | Y351* | NA | G | G/G | G/G | G/C | G/G | NA | NA | NA | NA | NA | NA |
| chr7 | 4304937 | rs117504728 | SDK1 | T2188K | 0.0096 | C | C/A | C/C | C/A | C/A | 7 | 310 | 39 | 736 | 0.03 | 0.41 |
| chr15 | 40916632 | rs141726041 | CASC5 | D1390E | 0.0066 | T | T/A | T/A | T/A | T/T | 14 | 336 | 16 | 736 | 0.07 | 1.96 |
| chr1 | 51826853 | rs151081025 | EPS15 | N845S | 0.0086 | T | T/C | T/C | T/C | T/T | 10 | 324 | 11 | 736 | 0.10 | 2.10 |
| chr2 | 234201922 | rs140671487 | ATG16L1 | A531V | 0.0050 | C | C/T | C/T | C/T | C/T | 9 | 338 | 26 | 526 | 0.11 | 0.53 |
| chr19 | 10736346 | rs201831788 | SLC44A2 | G13R | 0.0088 | G | G/A | G/A | G/A | G/A | 9 | 334 | 32 | 734 | 0.23 | 0.6 |
| chr1 | 24840882 | rs143770846 | RCAN3 | K7R | 0.0014 | A | A/G | A/G | A/G | A/A | 4 | 214 | 7 | 736 | 0.28 | 1.98 |
| chr16 | 21261289 | - | ANKS4B | R134S | NA | G | G/C | G/G | G/C | G/G | 1 | 212 | 0 | 366 | 0.37 | 5.20 |
| chr12 | 56365381 | rs2069413 | CDK2 | T256S | 0.0044 | C | C/G | C/G | C/G | C/C | 11 | 322 | 19 | 736 | 0.43 | 1.34 |
| chr3 | 64666931 | rs140444427 | ADAMTS9 | R209C | 0.0080 | G | G/A | G/G | G/A | G/G | 7 | 324 | 23 | 736 | 0.43 | 0.69 |
| chr11 | 35250725 | rs200125479 | CD44 | E649K | 0.0002 | G | G/A | G/G | G/A | G/A | 1 | 212 | 1 | 576 | 0.47 | 2.73 |
| chr1 | 214537946 | rs200340171 | PTPN14 | P1115L | 0.0032 | G | G/A | G/G | G/A | G/A | 6 | 338 | 14 | 526 | 0.49 | 0.66 |
| chr3 | 49947940 | - | MON1A | A428T | NA | C | C/T | C/C | C/T | C/T | 3 | 312 | 0 | 160 | 0.55 | NA |
| chr1 | 153651621 | - | NPR1 | R13S | NA | C | C/A | C/C | C/A | C/A | 2 | 202 | 1 | 318 | 0.56 | 3.17 |
| chr7 | 1588206 | rs150440402 | TMEM184A | V255I | 0.0026 | C | C/T | C/T | C/T | C/T | 7 | 322 | 13 | 736 | 0.63 | 1.24 |
| chr15 | 41148251 | rs116845898 | SPINT1 | G427S | 0.0050 | G | G/A | G/A | G/A | G/A | 5 | 318 | 16 | 736 | 0.64 | 0.72 |
| chr3 | 39166625 | rs148183642 | TTC21A | V397M | 0.0036 | G | G/A | G/G | G/A | G/A | 1 | 214 | 7 | 736 | 0.69 | 0.49 |
| chr17 | 19687216 | rs55730189 | ULK2 | G752R | 0.0058 | C | C/T | C/T | C/T | C/T | 9 | 326 | 19 | 736 | 0.84 | 1.07 |
| chr17 | 21202191 | rs33911218 | MAP2K3 | P11T | 0.0002 | C | C/A | C/A | C/A | C/A | 168 | 330 | 160 | 320 | 0.88 | 1.04 |
| chr12 | 1040408 | rs370272002 | RAD52 | R55H | NA | C | C/T | C/C | C/T | C/C | 1 | 212 | 0 | 160 | 1.00 | 2.28 |
| chr17 | 10398298 | rs140873918 | MYH1 | Q1806K | 0.0004 | G | G/T | G/T | G/T | G/T | 1 | 210 | 2 | 576 | 1.00 | 1.37 |
| chr16 | 28603585 | rs55845129 | SULT1A2 | K258N | 0.0016 | T | T/A | T/T | T/A | T/T | 2 | 212 | 8 | 736 | 1.00 | 0.87 |
| chr3 | 37670789 | rs147773336 | ITGA9 | R601C | 0.0004 | C | C/T | C/C | C/T | C/T | 1 | 214 | 4 | 526 | 1.00 | 0.61 |
| chr2 | 43452757 | rs201963284 | ZFP36L2 | H62Q | 0.0020 | A | A/C | A/A | A/C | A/A | 3 | 328 | 5 | 576 | 1.00 | 1.05 |
| chr5 | 1815983 | - | NDUFS6 | G110C | NA | G | G/T | G/T | G/T | G/G | NA | NA | NA | NA | NA | NA |
| chr17 | 10551887 | - | MYH3 | N241S | NA | T | T/C | T/C | T/C | T/C | NA | NA | NA | NA | NA | NA |
| chr11 | 498548 | - | RNH1 | L289F | NA | G | G/A | G/A | G/A | G/G | NA | NA | NA | NA | NA | NA |
| chr14 | 23549329 | - | ACIN1 | E463D | NA | C | C/G | C/G | C/G | C/G | NA | NA | NA | NA | NA | NA |
| chr11 | 27016120 | - | FIBIN | C16Y | NA | G | G/A | G/A | G/A | G/G | NA | NA | NA | NA | NA | NA |
| chr16 | 27454345 | rs146177564 | IL21R | R139C | 0.0024 | C | C/T | C/T | C/T | C/C | NA | NA | NA | NA | NA | NA |
| chr14 | 36004753 | - | INSM2 | H432L | NA | A | A/T | A/T | A/T | A/A | NA | NA | NA | NA | NA | NA |
| chr3 | 47883141 | - | DHX30 | A196T | NA | G | G/A | G/G | G/A | G/A | NA | NA | NA | NA | NA | NA |
| chr12 | 53897517 | - | TARBP2 | S116F | NA | C | C/T | C/T | C/T | C/C | NA | NA | NA | NA | NA | NA |
| chr14 | 57046739 | - | C14orf101 | V36A | NA | T | T/C | T/C | T/C | T/T | NA | NA | NA | NA | NA | NA |
| chr16 | 58757654 | rs138952206 | GOT2 | R81H | 0.0002 | C | C/T | C/C | C/T | C/T | NA | NA | NA | NA | NA | NA |
| chr15 | 59499363 | - | LDHAL6B | I75T | NA | T | T/C | T/C | T/C | T/T | NA | NA | NA | NA | NA | NA |
| chr20 | 60883146 | rs373828612 | ADRM1 | R309H | NA | G | G/A | G/G | G/A | G/A | NA | NA | NA | NA | NA | NA |
| chr14 | 62187265 | - | HIF1A | L67F | NA | G | G/T | G/T | G/T | G/G | NA | NA | NA | NA | NA | NA |
| chr16 | 68267770 | rs78705766 | ESRP2 | D153N | 0.0002 | C | C/T | C/C | C/T | C/T | NA | NA | NA | NA | NA | NA |
| chr16 | 69875980 | rs184547498 | WWP2 | R178W | 0.0002 | C | C/T | C/C | C/T | C/T | NA | NA | NA | NA | NA | NA |
| chr9 | 79634858 | - | FOXB2 | M96I | NA | G | G/T | G/G | G/T | G/G | NA | NA | NA | NA | NA | NA |
| chr11 | 102589280 | - | MMP8 | H217Y | NA | G | G/A | G/G | G/A | G/G | NA | NA | NA | NA | NA | NA |
| chr13 | 103446154 | - | KDELC1 | P131S | NA | G | G/A | G/G | G/A | G/G | NA | NA | NA | NA | NA | NA |
| chr2 | 107423238 | - | ST6GAL2 | N496D | NA | T | T/C | T/T | T/C | T/T | NA | NA | NA | NA | NA | NA |
| chr11 | 129753091 | - | NFRKB | D276G | NA | T | T/C | T/C | T/C | T/T | NA | NA | NA | NA | NA | NA |
| chr9 | 140289770 | rs374664490 | EXD3 | E14K | NA | C | C/T | C/C | C/T | C/T | NA | NA | NA | NA | NA | NA |
| chr6 | 145114993 | rs116739902 | UTRN | P2982S | 0.0012 | C | C/T | C/T | C/T | C/C | NA | NA | NA | NA | NA | NA |
| chr1 | 147380272 | - | GJA8 | V64I | NA | G | G/A | G/G | G/A | G/A | NA | NA | NA | NA | NA | NA |
| chr7 | 150644474 | rs373394254 | KCNH2 | R1032W | 0.0004 | G | G/A | G/G | G/A | G/G | NA | NA | NA | NA | NA | NA |
| chr1 | 161993059 | - | OLFML2B | N54K | NA | G | G/T | G/G | G/T | G/T | NA | NA | NA | NA | NA | NA |

Chr, chromosome; Position, physical position of the variant; SNP_ID, rsID in dbSNP (https://www.ncbi.nlm.nih.gov/snp/); MAF_1000Genomes, minor allele frequency in the dataset of the 1000 Genomes Project ^22,23^; Reference allele, allele of reference genome hg19 (GRCh37: https://grch37.ensembl.org/Homo_sapiens); AD_AC, allele count of AD cases; AD_AN, allele number of AD cases; ctrl_AC, allele count of the controls; ctrl_AN, allele number of the controls; *Fisher P*, Fisher exact *P*-value; OR, odds ratio.

107 patients with early onset familial AD (EOFAD) enrolled from South China (Yunnan, Hunan, and Sichuan provinces) ^16^ and 62 EOFAD cases collected from North China (Beijing, Hebei, and Shandong provinces) ^21^ were considered as the AD cases. The control (ctl) sample referred to the population control described in our previous study^16^, which contained 386 pooled Han Chinese individuals from different sources.

Table S3.

**KEGG pathway, GO CC (cellular component) and GO BP (biological processes) analyses of dysregulated genes in the HM and U251-APP cells in ACAA1 p.N299S group compared with ACAA1 WT group**

| **Cell Type** | **Type** | **ID** | **Description** | ***P-*value ^a^** | ***P*_adjust_ ^b^** | **FDR q-value ^c^** |
| --- | --- | --- | --- | --- | --- | --- |
| HM | KEGG | hsa04142 | Lysosome | 2.50E-04 | 9.06E-03 | 8.06E-03 |
| HM | KEGG | hsa04218 | Cellular senescence | 5.58E-04 | 1.27E-02 | 1.13E-02 |
| U251-APP | KEGG | hsa05010 | Alzheimer's Disease | 2.78E-20 | 3.05E-18 | 2.26E-18 |
| U251-APP | KEGG | hsa04142 | Lysosome | 6.96E-06 | 1.64E-04 | 1.21E-04 |
| U251-APP | KEGG | hsa04722 | Neurotrophin signaling pathway | 1.78E-03 | 1.41E-02 | 1.04E-02 |
| U251-APP | KEGG | hsa04218 | Cellular senescence | 4.83E-06 | 1.32E-04 | 9.82E-05 |
| U251-APP | KEGG | hsa04979 | Cholesterol metabolism | 5.99E-03 | 3.34E-02 | 2.48E-02 |
| U251-APP | GO-CC | GO:0043202 | Lysosomal lumen | 1.64E-06 | 2.49E-05 | 1.82E-05 |
| U251-APP | GO-CC | GO:0030426 | Growth cone | 4.10E-06 | 5.30E-05 | 3.88E-05 |
| U251-APP | GO-CC | GO:0099572 | Postsynaptic specialization | 9.44E-05 | 8.24E-04 | 6.03E-04 |
| U251-APP | GO-CC | GO:0098984 | Neuron to neuron synapse | 1.69E-05 | 1.74E-04 | 1.28E-04 |
| U251-APP | GO-CC | GO:0048786 | Presynaptic active zone | 1.09E-03 | 7.41E-03 | 5.43E-03 |
| U251-APP | GO-CC | GO:0150034 | Distal axon | 2.31E-03 | 1.39E-02 | 1.02E-02 |
| HM | GO-CC | GO:0014069 | Postsynaptic density | 1.74E-08 | 4.35E-07 | 3.39E-07 |
| HM | GO-CC | GO:0098984 | Neuron to neuron synapse | 9.25E-08 | 2.04E-06 | 1.59E-06 |
| HM | GO-CC | GO:0043202 | Lysosomal lumen | 3.67E-07 | 7.35E-06 | 5.73E-06 |
| HM | GO-CC | GO:0005765 | Lysosomal membrane | 8.58E-06 | 1.45E-04 | 1.13E-04 |
| HM | GO-CC | GO:0097386 | Glial cell projection | 1.03E-04 | 1.29E-03 | 1.00E-03 |
| HM | GO-CC | GO:0150034 | Distal axon | 2.66E-04 | 2.91E-03 | 2.27E-03 |
| HM | GO-CC | GO:0098685 | Schaffer collateral - CA1 synapse | 2.06E-03 | 1.65E-02 | 1.29E-02 |
| HM | GO-CC | GO:0043197 | Dendritic spine | 3.34E-03 | 2.47E-02 | 1.92E-02 |
| HM | GO-CC | GO:0044309） | Neuron spine | 4.23E-03 | 3.05E-02 | 2.38E-02 |
| HM | GO-CC | GO:0048786 | Presynaptic active zone | 4.59E-03 | 3.26E-02 | 2.54E-02 |
| U251-APP | GO-BP | GO:0007041 | Lysosomal transport | 6.62E-10 | 5.34E-08 | 4.57E-08 |
| U251-APP | GO-BP | GO:0008089 | Anterograde axonal transport | 1.00E-03 | 1.38E-02 | 1.18E-02 |
| U251-APP | GO-BP | GO:0098930 | Axonal transport | 3.03E-03 | 3.37E-02 | 2.89E-02 |
| U251-APP | GO-BP | GO:0006622 | Protein targeting to lysosome | 1.74E-03 | 2.16E-02 | 1.85E-02 |
| U251-APP | GO-BP | GO:0032226 | Positive regulation of synaptic transmission, dopaminergic | 2.17E-04 | 3.88E-03 | 3.32E-03 |
| U251-APP | GO-BP | GO:0150003 | Regulation of spontaneous synaptic transmission | 2.18E-04 | 3.88E-03 | 3.32E-03 |
| U251-APP | GO-BP | GO:1900272 | Negative regulation of long-term synaptic potentiation | 9.17E-04 | 1.28E-02 | 1.09E-02 |
| U251-APP | GO-BP | GO:0032225 | Regulation of synaptic transmission, dopaminergic | 3.91E-03 | 4.12E-02 | 3.53E-02 |
| U251-APP | GO-BP | GO:1900453 | Negative regulation of long-term synaptic depression | 2.04E-03 | 2.42E-02 | 2.07E-02 |
| U251-APP | GO-BP | GO:0031914 | Negative regulation of synaptic plasticity | 1.99E-02 | 1.35E-01 | 1.15E-01 |
| U251-APP | GO-BP | GO:1990090 | Cellular response to nerve growth factor stimulus | 4.11E-04 | 6.65E-03 | 5.70E-03 |
| U251-APP | GO-BP | GO:1990089 | Response to nerve growth factor | 4.55E-04 | 7.21E-03 | 6.17E-03 |
| U251-APP | GO-BP | GO:0090647 | Modulation of age-related behavioral decline | 7.65E-05 | 1.60E-03 | 1.37E-03 |
| U251-APP | GO-BP | GO:1901216 | Positive regulation of neuron death | 4.01E-05 | 9.41E-04 | 8.06E-04 |
| U251-APP | GO-BP | GO:0008654 | Phospholipid biosynthetic process | 1.05E-05 | 2.92E-04 | 2.50E-04 |
| U251-APP | GO-BP | GO:0044242 | Cellular lipid catabolic process | 6.80E-04 | 9.81E-03 | 8.40E-03 |
| U251-APP | GO-BP | GO:0006643 | Membrane lipid metabolic process | 3.71E-03 | 3.95E-02 | 3.38E-02 |
| U251-APP | GO-BP | GO:0046466 | Membrane lipid catabolic process | 4.34E-03 | 4.45E-02 | 3.81E-02 |
| U251-APP | GO-BP | GO:0034440 | Lipid oxidation | 6.88E-03 | 6.24E-02 | 5.34E-02 |
| U251-APP | GO-BP | GO:0006635 | Fatty acid beta-oxidation | 1.59E-04 | 3.00E-03 | 2.57E-03 |
| U251-APP | GO-BP | GO:0019395 | Fatty acid oxidation | 4.70E-03 | 4.77E-02 | 4.08E-02 |
| HM | GO-BP | GO:0050808 | Synapse organization | 3.09E-08 | 2.41E-06 | 2.03E-06 |
| HM | GO-BP | GO:0051963 | Regulation of synapse assembly | 1.77E-05 | 7.09E-04 | 5.97E-04 |
| HM | GO-BP | GO:0050803 | Regulation of synapse structure or activity | 2.28E-05 | 8.53E-04 | 7.19E-04 |
| HM | GO-BP | GO:0048167 | Regulation of synaptic plasticity | 3.07E-05 | 1.09E-03 | 9.20E-04 |
| HM | GO-BP | GO:0050807 | Regulation of synapse organization | 3.34E-05 | 1.18E-03 | 9.94E-04 |
| HM | GO-BP | GO:0035418 | Protein localization to synapse | 3.51E-05 | 1.22E-03 | 1.03E-03 |
| HM | GO-BP | GO:0007416 | Synapse assembly | 6.71E-05 | 2.02E-03 | 1.70E-03 |
| HM | GO-BP | GO:1902473 | Regulation of protein localization to synapse | 7.20E-05 | 2.11E-03 | 1.77E-03 |
| HM | GO-BP | GO:0050804 | Modulation of chemical synaptic transmission | 8.86E-05 | 2.51E-03 | 2.11E-03 |
| HM | GO-BP | GO:0051965 | Positive regulation of synapse assembly | 9.40E-05 | 2.63E-03 | 2.22E-03 |
| HM | GO-BP | GO:0099177 | Regulation of trans-synaptic signaling | 9.75E-05 | 2.71E-03 | 2.28E-03 |
| HM | GO-BP | GO:1903539 | Protein localization to postsynaptic membrane | 1.64E-04 | 4.08E-03 | 3.44E-03 |
| HM | GO-BP | GO:1905606 | Regulation of presynapse assembly | 3.01E-04 | 6.66E-03 | 5.61E-03 |
| HM | GO-BP | GO:0099174 | Regulation of presynapse organization | 4.56E-04 | 9.43E-03 | 7.95E-03 |
| HM | GO-BP | GO:0099054 | Presynapse assembly | 5.25E-04 | 1.03E-02 | 8.72E-03 |
| HM | GO-BP | GO:0098969 | Neurotransmitter receptor transport to postsynaptic membrane | 1.01E-03 | 1.72E-02 | 1.45E-02 |
| HM | GO-BP | GO:0048169 | Regulation of long-term neuronal synaptic plasticity | 1.27E-03 | 2.04E-02 | 1.72E-02 |
| HM | GO-BP | GO:0099172 | Presynapse organization | 1.28E-03 | 2.05E-02 | 1.72E-02 |
| HM | GO-BP | GO:1902474 | Positive regulation of protein localization to synapse | 1.40E-03 | 2.20E-02 | 1.85E-02 |
| HM | GO-BP | GO:0099173 | Postsynapse organization | 1.57E-03 | 2.44E-02 | 2.05E-02 |
| HM | GO-BP | GO:1903540 | Establishment of protein localization to postsynaptic membrane | 1.68E-03 | 2.56E-02 | 2.15E-02 |
| HM | GO-BP | GO:0007411 | Axon guidance | 4.16E-10 | 4.07E-08 | 3.43E-08 |
| HM | GO-BP | GO:0007409 | Axonogenesis | 5.23E-10 | 5.06E-08 | 4.26E-08 |
| HM | GO-BP | GO:0050770 | Regulation of axonogenesis | 1.72E-08 | 1.38E-06 | 1.16E-06 |
| HM | GO-BP | GO:0050771 | Negative regulation of axonogenesis | 1.37E-05 | 5.72E-04 | 4.82E-04 |
| HM | GO-BP | GO:0030516 | Regulation of axon extension | 1.87E-05 | 7.41E-04 | 6.24E-04 |
| HM | GO-BP | GO:0050772 | Positive regulation of axonogenesis | 9.81E-05 | 2.71E-03 | 2.28E-03 |
| HM | GO-BP | GO:0048675 | Axon extension | 1.55E-04 | 3.99E-03 | 3.36E-03 |
| HM | GO-BP | GO:0030517 | Negative regulation of axon extension | 8.48E-04 | 1.52E-02 | 1.28E-02 |
| HM | GO-BP | GO:0036515 | Serotonergic neuron axon guidance | 1.29E-03 | 2.05E-02 | 1.72E-02 |
| HM | GO-BP | GO:1902667 | Regulation of axon guidance | 2.03E-03 | 2.96E-02 | 2.50E-02 |
| HM | GO-BP | GO:0019896 | Axonal transport of mitochondrion | 2.96E-03 | 3.91E-02 | 3.30E-02 |
| HM | GO-BP | GO:0048841 | Regulation of axon extension involved in axon guidance | 3.12E-03 | 4.07E-02 | 3.43E-02 |
| HM | GO-BP | GO:0008366 | Axon ensheathment | 3.16E-03 | 4.10E-02 | 3.45E-02 |
| HM | GO-BP | GO:0071398 | Cellular response to fatty acid | 9.65E-04 | 1.68E-02 | 1.41E-02 |
| HM | GO-BP | GO:0070542 | Response to fatty acid | 1.50E-03 | 2.34E-02 | 1.97E-02 |
| HM | GO-BP | GO:0007611 | Learning or memory | 8.57E-05 | 2.44E-03 | 2.05E-03 |
| HM | GO-BP | GO:0007613 | Memory | 2.11E-03 | 3.06E-02 | 2.58E-02 |
| HM | GO-BP | GO:0097485 | Neuron projection guidance | 2.25E-10 | 2.25E-08 | 1.90E-08 |
| HM | GO-BP | GO:0010977 | Negative regulation of neuron projection development | 9.41E-07 | 5.54E-05 | 4.67E-05 |
| HM | GO-BP | GO:0045665 | Negative regulation of neuron differentiation | 1.51E-06 | 8.50E-05 | 7.16E-05 |
| HM | GO-BP | GO:0010976 | Positive regulation of neuron projection development | 1.97E-06 | 1.07E-04 | 9.02E-05 |
| HM | GO-BP | GO:0045666 | Positive regulation of neuron differentiation | 3.12E-06 | 1.56E-04 | 1.32E-04 |
| HM | GO-BP | GO:1990138 | Neuron projection extension | 2.40E-05 | 8.83E-04 | 7.44E-04 |
| HM | GO-BP | GO:0021954 | Central nervous system neuron development | 6.59E-05 | 2.00E-03 | 1.69E-03 |
| HM | GO-BP | GO:0051402 | Neuron apoptotic process | 4.66E-04 | 9.60E-03 | 8.09E-03 |
| HM | GO-BP | GO:0043524 | Negative regulation of neuron apoptotic process | 5.79E-04 | 1.13E-02 | 9.53E-03 |
| HM | GO-BP | GO:0048169 | Regulation of long-term neuronal synaptic plasticity | 1.27E-03 | 2.04E-02 | 1.72E-02 |
| HM | GO-BP | GO:0036515 | Serotonergic neuron axon guidance | 1.29E-03 | 2.05E-02 | 1.72E-02 |
| HM | GO-BP | GO:0021953 | Central nervous system neuron differentiation | 1.34E-03 | 2.12E-02 | 1.78E-02 |
| HM | GO-BP | GO:0043523 | Regulation of neuron apoptotic process | 1.58E-03 | 2.45E-02 | 2.06E-02 |
| HM | GO-BP | GO:0070997 | Neuron death | 2.06E-03 | 3.00E-02 | 2.53E-02 |
| HM | GO-BP | GO:0001764 | Neuron migration | 2.50E-03 | 3.51E-02 | 2.95E-02 |
| HM | GO-BP | GO:0007272 | Ensheathment of neurons | 3.16E-03 | 4.10E-02 | 3.45E-02 |

KEGG, Kyoto Encyclopedia of Genes and Genomes; GO-CC, gene ontology-cellular component; GO-BP, gene ontology-biological processes; FDR: false discovery rate.

**^a^** *P*-value of the enrichment analysis calculated by hypergeometric test

**^b^** *P*_adjust_, the BH-adjusted *P*-value, was calculated by adjusted *P*-value with Benjamini-Hochberg (BH) method

**^c^** FDR q-value was calculated by adjusted *P-*value with an optimized false discovery rate (FDR) approach

Table S4.

**Primary and secondary antibodies and chemicals used in this study**

| **Antibodies** | **Source** | **Catalog no.** | **Western blot** | **IF** | **IHC** |
| --- | --- | --- | --- | --- | --- |
| ***Primary antibody*** |  |  |  |  |  |
| Rabbit monoclonal anti-GluR1 (phospho S381) | Abcam | ab109464 | 1:1000 | - | - |
| Rabbit polyclonal anti-GluR1 | Elabscience | E-AB-31551 | 1:1000 | - | - |
| Rabbit polyclonal anti-GRIN2B | Elabscience | E-AB-15807 | 1:1000 | - | - |
| Rabbit polyclonal anti-LAMP1 | Abcam | ab24170 | 1:1000 | - | - |
| Rabbit polyclonal anti-CTSB | Affinity Bioscience | DF6149 | 1:1000 | - | - |
| Rabbit monoclonal ACAA1 | Cell Signaling Technology | 12210 | 1:1000 | - | - |
| Rabbit polyclonal anti-CTSD | Cell Signaling Technology | DF6486 | 1:1000 | - | - |
| Rabbit polyclonal anti-PSD95 | Abcam | ab18258 | 1:1000 | - | - |
| Rabbit polyclonal anti-TFEB | Cell Signaling Technology | 4240 | 1:1000 | - | - |
| Rabbit monoclonal anti-NeuN | Abcam | ab177487 | 1:1000 | 1:500 | - |
| Mouse monoclonal anti-IBA1 | Millipore | MABN92 | - | 1:300 | - |
| Mouse monoclonal anti-Flag-Tag | EnoGene | E12-001 | 1:10000 | - | - |
| Rabbit monoclonal anti-LAMP2A | Abcam | ab125068 | 1:1000 | - | - |
| Mouse monoclonal anti-Synaptophysin | Millipore | MAB5258-50UG | 1:10000 | - | - |
| Mouse monoclonal anti-GFP | EnoGene | E12-009 | 1:1000 | - | - |
| Chicken polyclonal anti-GFP | Abcam | ab13970 | - | 500 | - |
| Rabbit polyclonal anti-MAP1LC3/LC3 | Proteintech | 14600-1-AP | 1:1000 | - | - |
| Rabbit polyclonal anti-SQSTM1 | Elabscience | EAP3350 | 1:1000 | - | - |
| Rabbit monoclonal anti anti-PEN2 | Cell Signaling Technology | 8598 | 1:1000 | - | - |
| Rabbit monoclonal anti-PSEN1 | Cell Signaling Technology | 5643 | 1:1000 | - | - |
| Rabbit monoclonal anti-PSEN2 | Cell Signaling Technology | 9979 | 1:1000 | - | - |
| Rabbit monoclonal anti-BACE1 | Cell Signaling Technology | 5606 | 1:1000 | - | - |
| Mouse anti-β-amyloid,17–24(4G8) | BioLegend | 800701 | - | 1:500 | 1:500 |
| Mouse monoclonal anti-β-tubulin | EnoGene | E1C601 | 1:10000 | - | - |
| Mouse monoclonal anti-GAPDH | Proteintech | 60004-1-Ig | 1:10000 | - | - |
| Mouse monoclonal anti-ACTB | Beijing Zhong Shan-Golden Bridge Biological Technology CO., LTD | TA-09 | 1:10000 | - | - |
| ***Secondary antibody*** |  |  |  |  |  |
| Peroxidase-conjugated anti-rabbit antibody | KPL | 474-1516 | 1:10000 | - | - |
| Peroxidase-conjugated anti-mouse antibody | KPL | 474-1806 | 1:10000 | - | - |
| Alexa Fluor® 594-conjugated anti-rabbit IgG | Invitrogen | A21207 | - | 1:500 | - |
| Alexa Fluor® 488-conjugated anti-chicken IgG | Invitrogen | A11039 | - | 1:500 | - |
| Alexa Fluor® 555 anti-mouse IgG | Invitrogen | A31570 | - | 1:500 | - |
| HRP-conjugated goat anti-mouse antibody | Servicebio | GB23301 | - | - | 1:200 |
| ***Chemicals*** | Source | Catalog no. | - | - | - |
| 4', 6-diamidino-2-phenylindole (DAPI) | Roche | 10236276001 | - | - | - |
| Bafilomycin A1 (BAFA1) | InvivoGen | tlrl-baf | - | - | - |
| NH4CL | Sigma | A9434 | - | - | - |
| Pentobarbital | Sigma | P3761 | - | - | - |

IF - immunofluorescence; IHC - Immunohistochemistry

**Reference**

1 Zhang, D. F. *et al.* CFH variants affect structural and functional brain changes and genetic risk of Alzheimer's disease. *Neuropsychopharmacol* **41**, 1034-1045 (2016).

2 Xiang, Q. *et al.* Rare genetic variants of the Transthyretin gene are associated with Alzheimer's disease in Han Chinese. *Mol Neurobiol* **54**, 5192-5200 (2017).

3 Kumar, S., Stecher, G. & Tamura, K. MEGA7: molecular evolutionary genetics analysis version 7.0 for bigger datasets. *Mol Biol Evol* **33**, 1870-1874 (2016).

4 Xu, M. *et al.* A systematic integrated analysis of brain expression profiles reveals YAP1 and other prioritized hub genes as important upstream regulators in Alzheimer's disease. *Alzheimers Dement* **14**, 215-229 (2018).

5 Bolger, A. M., Lohse, M. & Usadel, B. Trimmomatic: a flexible trimmer for Illumina sequence data. *Bioinformatics* **30**, 2114-2120 (2014).

6 Dobin, A. *et al.* STAR: ultrafast universal RNA-seq aligner. *Bioinformatics* **29**, 15-21 (2013).

7 Liao, Y., Smyth, G. K. & Shi, W. The Subread aligner: fast, accurate and scalable read mapping by seed-and-vote. *Nucleic Acids Res* **41**, e108 (2013).

8 Love, M. I., Huber, W. & Anders, S. Moderated estimation of fold change and dispersion for RNA-seq data with DESeq2. *Genome Biol* **15**, 550 (2014).

9 The Gene Ontology, C. The Gene Ontology Resource: 20 years and still GOing strong. *Nucleic Acids Res* **47**, D330-D338 (2019).

10 Kanehisa, M., Furumichi, M., Tanabe, M., Sato, Y. & Morishima, K. KEGG: new perspectives on genomes, pathways, diseases and drugs. *Nucleic Acids Res* **45**, D353-D361 (2017).

11 Yu, G., Wang, L. G., Han, Y. & He, Q. Y. clusterProfiler: an R package for comparing biological themes among gene clusters. *OMICS* **16**, 284-287 (2012).

12 Subramanian, A. *et al.* Gene set enrichment analysis: a knowledge-based approach for interpreting genome-wide expression profiles. *Proc Natl Acad Sci U S A* **102**, 15545-15550 (2005).

13 Luo, R. *et al.* Activation of PPARA-mediated autophagy reduces Alzheimer disease-like pathology and cognitive decline in a murine model. *Autophagy* **16**, 52-69 (2020).

14 Su, L. Y. *et al.* Atg5- and Atg7-dependent autophagy in dopaminergic neurons regulates cellular and behavioral responses to morphine. *Autophagy* **13**, 1496-1511 (2017).

15 Sheng, N., Shi, Y. S. & Nicoll, R. A. Amino-terminal domains of kainate receptors determine the differential dependence on Neto auxiliary subunits for trafficking. *Proc Natl Acad Sci U S A* **114**, 1159-1164 (2017).

16 Zhang, D. F. *et al.* Complement C7 is a novel risk gene for Alzheimer's disease in Han Chinese. *Natl Sci Rev* **6**, 257-274 (2019).

17 Fu, A. K. Y. *et al.* IL-33 ameliorates Alzheimer's disease-like pathology and cognitive decline. *P Natl Acad Sci USA* **113**, E2705-E2713 (2016).

18 Alves, S. *et al.* Interleukin-2 improves amyloid pathology, synaptic failure and memory in Alzheimer's disease mice. *Brain* **140**, 826-842 (2017).

19 Martinez Hernandez, A. *et al.* The diphenylpyrazole compound anle138b blocks Abeta channels and rescues disease phenotypes in a mouse model for amyloid pathology. *EMBO Mol Med* **10**, 32-47 (2018).

20 Krauthausen, M. *et al.* CXCR3 promotes plaque formation and behavioral deficits in an Alzheimer's disease model. *J Clin Invest* **125**, 365-378 (2015).

21 Wang, G. *et al.* Mutation and association analyses of dementia-causal genes in Han Chinese patients with early-onset and familial Alzheimer's disease. *J Psychiatr Res* **113**, 141-147 (2019).

22 Sudmant, P. H. *et al.* An integrated map of structural variation in 2,504 human genomes. *Nature* **526**, 75-81 (2015).

23 Genomes Project, C. *et al.* A global reference for human genetic variation. *Nature* **526**, 68-74 (2015).

24 Kumar, P., Henikoff, S. & Ng, P. C. Predicting the effects of coding non-synonymous variants on protein function using the SIFT algorithm. *Nat Protoc* **4**, 1073-1081 (2009).

25 Adzhubei, I. A. *et al.* A method and server for predicting damaging missense mutations. *Nat Methods* **7**, 248-249 (2010).

26 Adzhubei, I., Jordan, D. M. & Sunyaev, S. R. Predicting functional effect of human missense mutations using PolyPhen-2. *Curr Protoc Hum Genet* **Chapter 7**, Unit7 20 (2013).

27 Chun, S. & Fay, J. C. Identification of deleterious mutations within three human genomes. *Genome Res* **19**, 1553-1561 (2009).

28 Schwarz, J. M., Cooper, D. N., Schuelke, M. & Seelow, D. MutationTaster2: mutation prediction for the deep-sequencing age. *Nat Methods* **11**, 361-362 (2014).
